# An exceptional series of phase transitions in hydrophobic amino acids with linear side chains

**DOI:** 10.1107/S2052252516010472

**Published:** 2016-08-09

**Authors:** Carl Henrik Görbitz, Pavel Karen, Michal Dušek, Václav Petříček

**Affiliations:** a Department of Chemistry, University of Oslo, N-0315 Oslo, Norway; b Institute of Physics, Academy of Sciences of the Czech Republic, Na Slovance 2, 182 21 Praha 8, Czech Republic

**Keywords:** amino acids, disorder, hydrogen bonding, modulated phases, phase transitions, side-chain stacking, polymorphism, molecular crystals

## Abstract

The solid-state phase transitions and intermediate structures of *S*-2-amino­butanoic acid (l-2-aminobutyric acid), *S*-2-aminopentanoic acid (l-norvaline), *S*-2-aminohexanoic acid (l-norleucine) and l-methionine between 100 and 470 K, identified by differential scanning calorimetry, have been characterized in a comprehensive single-crystal X-ray diffraction investigation. Unlike other enantiomeric amino acids investigated until now, this group featuring linear side chains displays up to five distinct phases. The multiple transitions between them involve a number of different processes: alteration of the hydrogen-bond pattern, to our knowledge the first example of this observed for an amino acid, sliding of molecular bilayers, seen previously only for racemates and quasiracemates, concerted side-chain rearrangements and abrupt as well as gradual modifications of the side-chain disorder. Ordering of l-norleucine upon cooling even proceeds *via* an incommensurately modulated structure. l-Methionine has previously been described as being fully ordered at room temperature. An accurate refinement now reveals extensive disorder for both molecules in the asymmetric unit, while two previously unknown phases occur above room temperature.

## Abstract 2

Four amino acids with linear, hydrophobic side chains display an unprecedented series of solid-state phase transitions between 100 and 470 K. These involve hydrogen-bond rearrangements, sliding of molecular bilayers in one or two dimensions and development of side-chain disorder. The first modulated structures of amino acids are reported.

## 1. Introduction   

The crystal structures of amino acids with hydrophobic side chains invariably incorporate molecular bilayers, each with a hydrogen-bonded core and two surfaces shaped by the amino acid side chains. A series of unique solid-state phase transitions, involving sliding along the hydrophobic interfaces between adjacent bilayers, have been observed for dl-methionine (Met; Mathieson, 1952 ▸; Taniguchi *et al.*, 1980 ▸; Alagar *et al.*, 2005 ▸; Görbitz *et al.*, 2014 ▸, 2015 ▸; Görbitz, 2014 ▸), dl-2-aminobutyric acid (Abu; Görbitz *et al.*, 2012 ▸; Nakata *et al.*, 1980 ▸; Voogd & Derissen, 1980 ▸; Akimoto & Iitaka, 1972 ▸; Ichikawa & Iitaka, 1968 ▸), dl-norvaline (Nva; Görbitz, 2011 ▸) and dl-norleucine (Nle; Coles *et al.*, 2009 ▸; Harding *et al.*, 1995 ▸; Dalhus & Görbitz, 1996*b* ▸; Smets *et al.*, 2015 ▸; van den Ende & Cuppen, 2014 ▸) as well as for the four quasiracemates l-Nva:d-Nle, l-Nva:d-Met, l-Nle:d-Met (Görbitz & Karen, 2015 ▸) and l-Abu:d-Met (Görbitz *et al.*, 2016 ▸). Little is known by comparison about the corresponding enantiomeric amino acids. Only one crystal form is known for l-Met, which has been studied at room temperature (Torii & Iitaka, 1973 ▸), 150 K (Sadler *et al.*, 2005 ▸) and 120 K (Dalhus & Görbitz, 1996*a* ▸). For l-Abu we previously found two forms at 110 K, both with four molecules in the asymmetric unit (*Z*′ = 4; Görbitz, 2010 ▸). Only a single structure (at room temperature) is available for l-Nle (Torii & Iitaka, 1973 ▸). l-Nva has not been subject to X-ray diffraction investigations in the past.
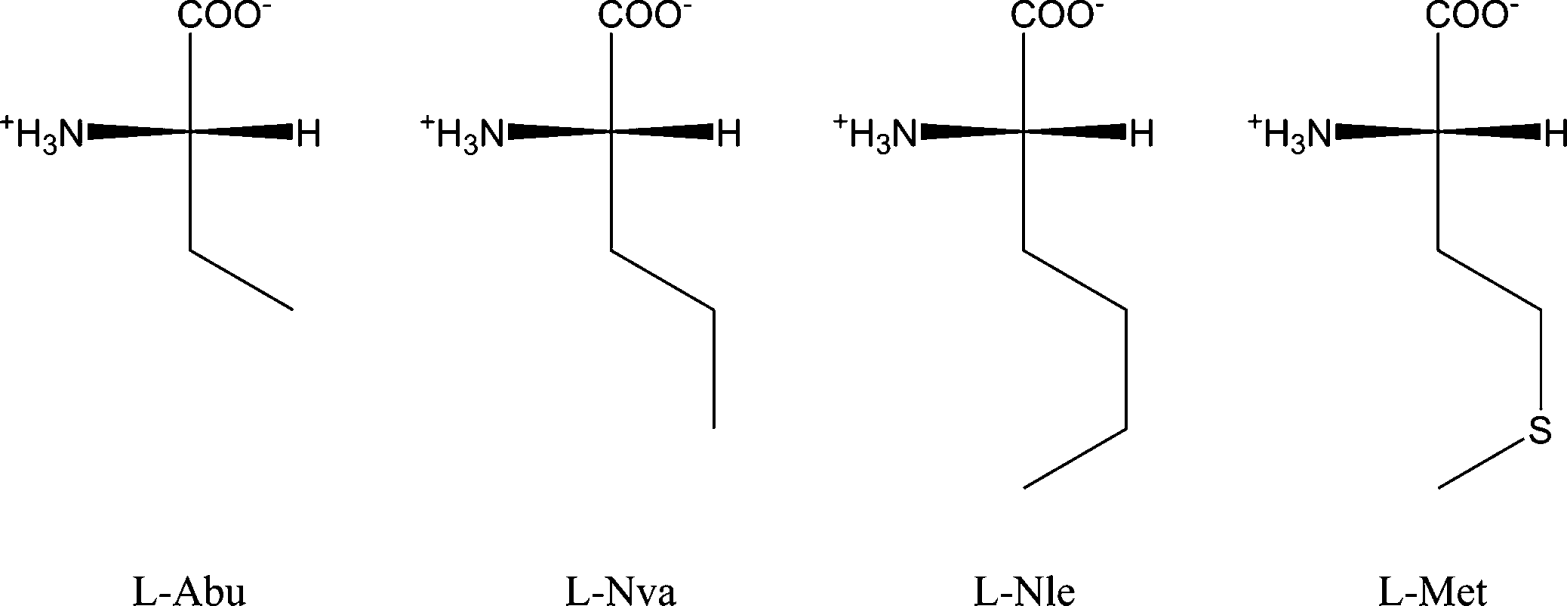


In the anticipation that temperature-induced phase transitions of the four amino acids with linear, hydrophobic side chains may not be limited to just the racemates, we have undertaken a systematic investigation of the enantiomeric substances in the temperature range between 100 and 470 K. We report here 18 single-crystal structures, refined from X-ray diffraction data collected at temperatures above and below transition temperatures recorded by differential scanning calorimetry (DSC).

## 2. Experimental   

### 2.1. Materials   

The amino acids l-Abu, l-Nva, l-Nle and l-Met were purchased from Sigma and used as received.

### 2.2. Crystal growth   

For each amino acid approximately 0.3 mg was dissolved in 30 µL of water in a 30 × 6 mm test tube and sealed with parafilm. A small hole was then pricked in the parafilm and the tube placed inside a larger test tube filled with 2 ml of acetonitrile. The system was capped and left for 5 d at 20°C. Thin platelets crystallized as the organic solvent diffused into the aqueous solutions.

### 2.3. Differential Scanning Calorimetry (DSC) measurements   

A liquid-nitrogen operated Perkin–Elmer Pyris 1 instrument was used to perform heating and cooling DSC cycles for ∼ 30 mg of the dried crystalline powders, sealed in aluminium pans of 30 µL volume, upon a decrease in scanning rate from 40 to 20 to 10 K min^−1^. The instrument was calibrated against the enthalpy and temperature standards of *n*-dodecane, *m*-nitrotoluene, *p*-nitrotoluene and indium of higher than 99.7% purity. Transition temperatures were evaluated by extrapolating peak-top temperatures to the zero scanning rate, transition enthalpies and entropies *via* integrating heat-flow peaks *versus* time as described by Karen (2003 ▸).

### 2.4. X-ray data collection and structure solution   

Single-crystal X-ray data collections were carried out with *APEX*2 software (Bruker, 2014 ▸) on a D8 Venture single-crystal CCD diffractometer equipped with an Oxford Cryosystems Cryostream *Plus* cooling unit and Mo *K*α radiation (λ = 0.71069 Å). Data sets were collected on cooling and heating from room temperature, except that data for l-Nle at 180 K were collected after first cooling the crystal to 100 K. Data integration and cell refinement were performed with *SAINT-Plus* (Bruker, 2014 ▸) with subsequent absorption correction by *SADABS* (Bruker, 2014 ▸) and structure solution with *SHELXLT* (Sheldrick, 2015*a* ▸). All structures are monoclinic and have a *b*-axis close to 5.2 Å. There is always a second axis of 9.5–9.6 Å in length, which, in order to facilitate comparison between the different structures, is here always taken to be the *a*-axis. The remaining *c*-axis is more variable (13–23 Å). Consequently, the unconventional space group *I*2 (rather than *C*2) is used for l-Nva at 220 K; analogous to the β form of l-Abu at 110 K (Görbitz, 2010 ▸).

### 2.5. Structure refinement   

Least-squares refinements were carried out with *SHELXL* (Sheldrick, 2015*b* ▸) against *F* ^2^. All structures except l-Nva at 100 and 190 K exhibit disorder, which becomes quite extensive above 300 K, making it tedious to match geometrically reasonable side-chain orientations with the electron density maps. Usually the whole molecule was regarded as disordered, but in some cases just one set of coordinates was used for the amino and carboxylate groups; for example, for conformations with very low occupancies (l-Nva at 220 K, l-Met at 293 and 320 K) or when the data-to-parameter ratio would otherwise be unduly compromised (l-Nle and l-Met at 405 K). C atoms with occupancy > 0.30 were refined anisotropically, with a single set of displacement parameters (through *SHELX* EADP commands) for positions of equivalent atoms closer than about 0.7 Å in order to avoid unwanted correlations. For the two 405 K structures, fixed isotropic *U* values of 0.30 Å^2^ were used for the terminal methyls. The covalent geometries of the independent disorder parts (up to four were observed) were generally linked by *SHELXL* SAME 0.004 0.006 commands to restrain bonds to be similar within an effective standard deviation of 0.004 Å and 1,3-distances [*d*(*X*1⋯*X*3) in the fragment *X*1—*X*2—*X*3, *X* = C/N/O] within 0.006 Å; effectively putting restraints on covalent angles. Furthermore, at 405 K the four atoms defining a *trans* torsion angle were restrained to be coplanar within a 0.05 Å or 0.07 Å standard deviation (*SHELXL* FLAT command) and 1,2-, 1,3- and 1,4-distances were restrained to preset values. Details are in the CIF files in the supporting information.

Crystal data and refinement results are listed in Tables 1–4 ▸ ▸ ▸ ▸, while a summary of the conformational disorder in terms of refined rotamers and their occupancies are given in Table 5 ▸. Complete lists of torsions angles and hydrogen-bond geometries are provided as supporting information, Tables S1 and S2. Illustrations of molecules and their crystal packing arrangements were prepared by *Mercury* (Macrae *et al.*, 2008 ▸).

### 2.6. Refinement of modulated phases   

Least-squares refinements for l-Nle were carried out with *JANA*2006 (Petříček *et al.*, 2014 ▸) against *F* ^2^. For comparison, both incommensurate and commensurate refinements were performed on each modulated phase. A so-called rigid-body modulation approach was used to fix the molecular geometry of each conformer at all its positions and occupancies generated from their complementing modulation waves on the periodicity refined in the (3 + 1)-dimensional superspace with either harmonic (Fourier series) or crenel (Petříček *et al.*, 1995 ▸) functions. Experimental and refinement details are provided in the supporting information, Tables S3 and S4.

## 3. Results and discussion   

### 3.1. Phase transitions between 100 and 470 K from DSC   

**Figure 1:**
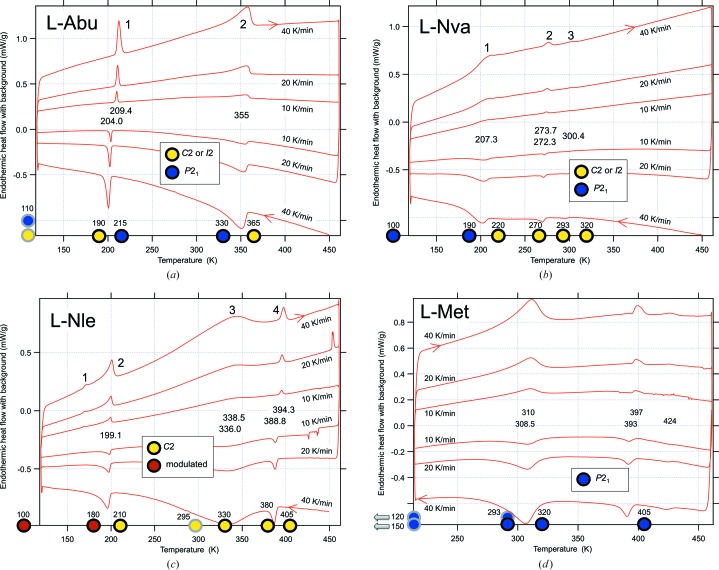
Cyclic DSC scans for l-Abu, l-Nva, l-Nle and l-Met at 40, 20 and 10 K min^−1^. The transition temperatures refer to peak maxima extrapolated to zero scanning rate. Transitions are numbered according to the description in the text. Circles at the bottom axis indicate temperatures for the single-crystal data collections. A grey outline is used for previous investigations for l-Abu (Görbitz, 2010 ▸), l-Nle (Torii & Iitaka, 1973 ▸) and l-Met (Torii & Iitaka, 1973 ▸; Sadler *et al.*, 2005 ▸; Dalhus & Görbitz, 1996*a* ▸).

The results from the DSC analysis are summarized in Table 6 ▸ and Fig. 1 ▸, a schematic illustration is shown in the supporting information, Fig. S1. Upon warming, two reproducible endothermic events are recorded for l-Abu. The first is sharp, while the second develops gradually from 270 K to a peak at 356 K and ceases abruptly around 360 K. For l-Nva transition 1 is rather broad, while transition 3 is very weak. Regarding transition 2 at 273 K, repeated measurement with a carefully dried sample confirmed the absence of water. Four reproducible endothermic events take place for l-Nle. The first (observable only upon heating) and second events overlap. Finally, for l-Met, three endothermic events are recorded between 100 and 470 K. The first is broad and develops between 250 and 340 K.

### 3.2. l-Abu   

**Figure 2:**
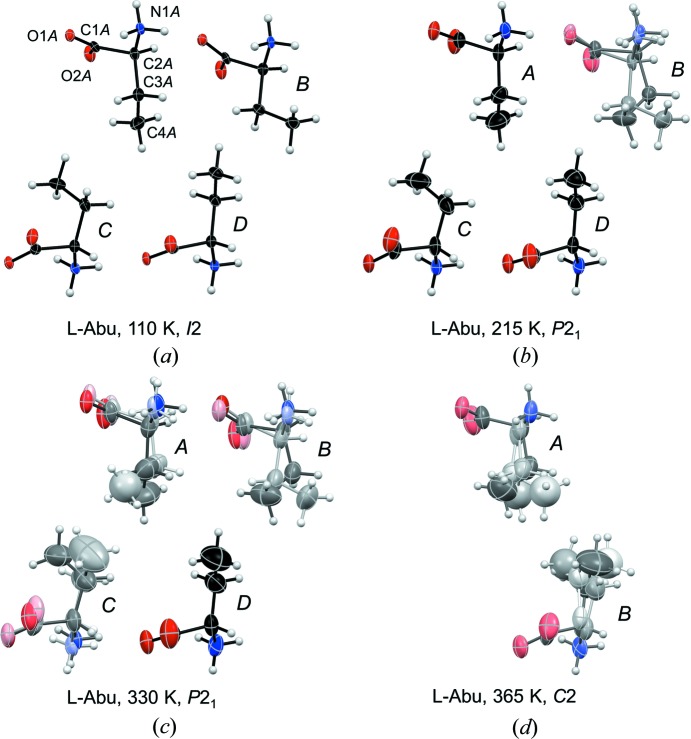
Molecular structures of l-Abu at (*a*) 110 (Görbitz, 2010 ▸), (*b*) 215, (*c*) 330 and (*d*) 365 K. Individual molecules in the asymmetric unit are labelled in italics (*A*, *B*, …) with illustrative atomic numbering given for one molecule. Thermal displacement ellipsoids are shown at the 50% probability level. Atom color depths reflect the occupancy of each conformation as listed in Table 5 ▸. The asymmetric unit of the *P*2_1_ form of l-Abu at 110 K looks exactly as the *I*2 asymmetric unit shown in (*a*).

A *P*2_1_ α form and a *I*2 β form, both with *Z*′ = 4 (Fig. 2 ▸ *a*), were previously identified for l-Abu at 110 K (Görbitz, 2010 ▸). We have now heated and cooled a selected crystal a number of times across the transition temperature at ∼ 207 K. Unit-cell determinations at 190 K always reproduced the β form, while the α form prevailed at 215 K. Accordingly, transition 1 in Fig. 1 ▸ represents a full and reversible conversion between the two l-Abu polymorphs. From this we conclude that the α form is unstable below transition 1, and that the *P*2_1_ crystal previously investigated at 110 K (Görbitz, 2010 ▸), the only one with this symmetry out of about ten tested, represented a freak occurrence of a specimen that failed to undergo the normal phase transition upon cooling.

**Figure 3:**
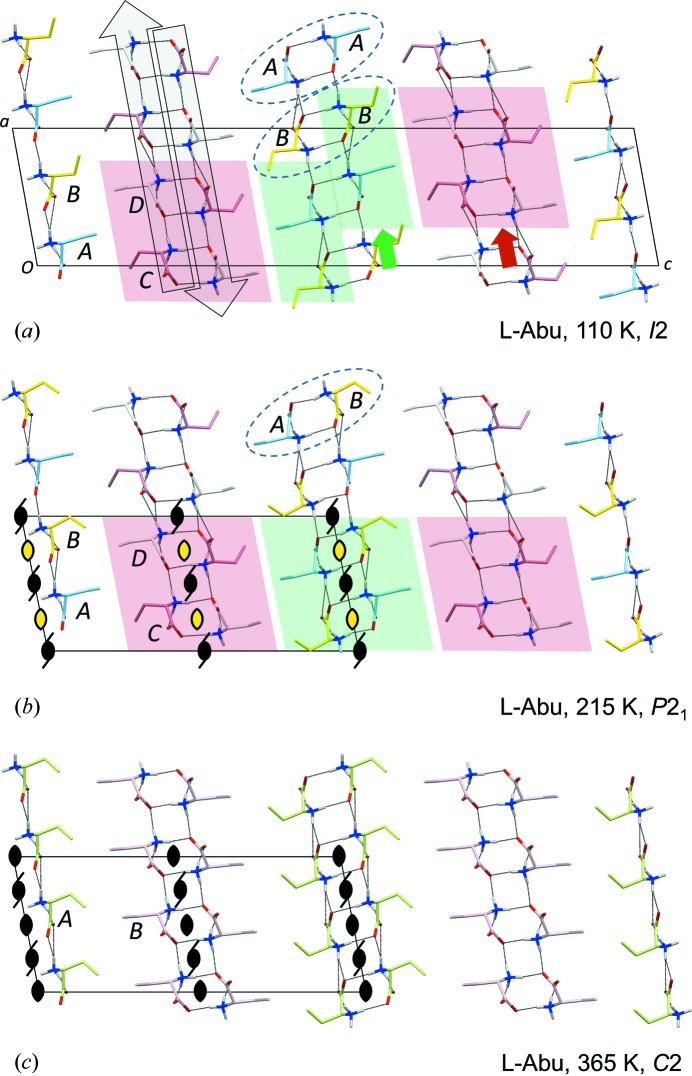
Crystal packing of l-Abu viewed along the *b* axis at (*a*) 110 K (Görbitz, 2010 ▸), (*b*) 215 K and (*c*) 365 K. In (*a*) and (*b*) C atoms of molecules *A*, *B*, *C* and *D* are colored in sky blue, yellow, pink and white, respectively. In (*c*) molecule *A* (average of *A* and *B* at lower temperatures) is olive green, while molecule *B* (average of *C* and *D* at lower temperatures) is light pink. Only the most populated side-chain conformation is shown for disordered molecules. Colored arrows in (*a*) highlight what may look like a slide at the center of the green bilayer and the full actual slide of the red bilayer, while large open arrows cover two antiparallel hydrogen-bonded sheets that constitute a hydrophilic layer at the core of the two molecular bilayers identified by red and green shades. Dashed ellipses show hydrogen-bonded amino acid dimers. Symmetry elements are included in (*b*) and (*c*), with yellow fill for pseudo-twofold rotation axes, see text for details.

**Figure 4:**
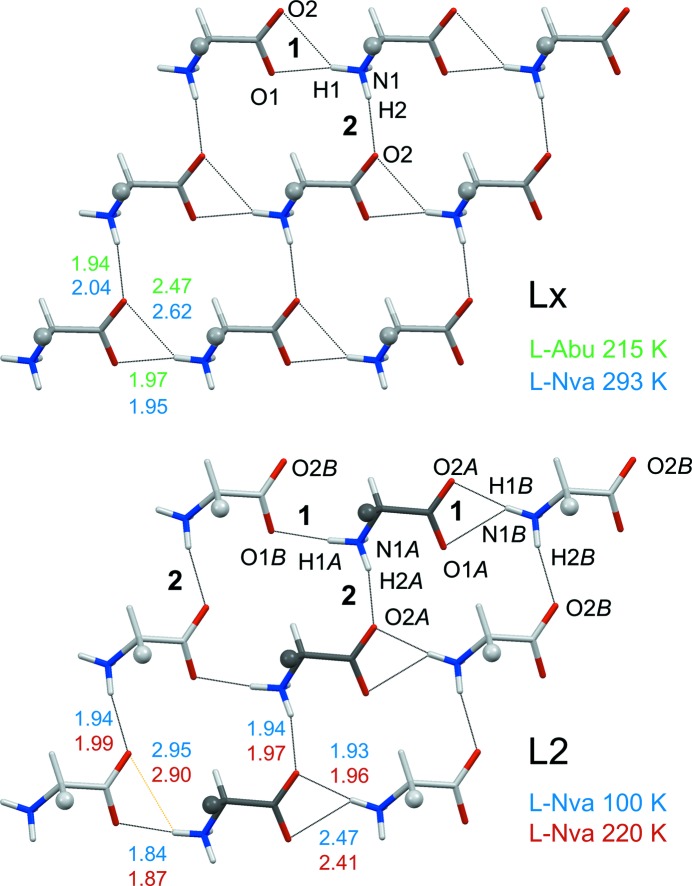
Hydrogen-bonded sheet of type Lx (Görbitz *et al.*, 2009 ▸) of l-Abu at 215 K and l-Nva at 293 K (top) and type L2 of l-Nva at 100 and 220 K (bottom). Side chains are shown as small spheres. Hydrogen bonds accepted by *syn* and *anti* carboxylate lone pairs are marked **1** and **2**, respectively; distances (in Å) for l-Abu are average values for four molecules. In the L2 sheet, the H1*B* of molecule *B* (light grey C atoms) is involved in a three-centered interaction to the carboxylate group of molecule *A* (dark grey C atoms), with H1*B*⋯O2*A* as the shorter contact, while H1*A* participates in a two-centered interaction to O1*B* (H1*A*⋯O2*B* in orange is > 2.8 Å). In the Lx sheet all *syn* (**1**) hydrogen bonds are similar, with H1⋯O1 as the shorter component of a three-centered interaction.

The packing diagrams in Fig. 3 ▸ show the typical construction of a crystal of a hydrophobic amino acid with stacking of molecular bilayers, each with two hydrogen-bonded sheets in its core. Four different types of sheets have been observed for enantiomeric amino acids (Görbitz *et al.*, 2009 ▸). The one observed here (Fig. 4 ▸) is called Lx (see the supporting information for notation). Notably, all molecules of such a sheet take part in equivalent intermolecular interactions, even when *Z*′ > 1 (here *Z*′ = 4).

A special property of the *I*2 and the *P*2_1_ l-Abu polymorphs, seen among previous amino acid structures only for form (I) (Ihlefeldt *et al.*, 2014 ▸) and form (III) (Mossou *et al.*, 2014 ▸) of l-phenylalanine, is the presence of two distinct types of molecular bilayers, one constructed from *A* and *B* molecules and one from *C* and *D* molecules. The latter is unperturbed by the phase change (Figs. 3 ▸ *a* and *b*), but some subtle yet intriguing changes occur for the *AB*-bilayers as only *A*:::*B* heterodimers are present in the *P*2_1_ structure, while the *I*2 structure has *A*:::*A* and *B*:::*B* homodimers. It would be unreasonable to assume that transitions between the two forms take place by sliding *inside* the hydrophilic layer (green arrow in Fig. 3 ▸ *a*), as this would require that all interactions were broken between the two Lx sheets (large open arrows) that constitute the Lx–Lx hydrogen-bonded layer. The mechanism must instead involve a combination of sliding every second molecular bilayer (observed previously for the intermediate phases of twin displacive transitions in some amino-acid quasiracemates; Görbitz & Karen, 2015 ▸) with concerted conformational changes for all amino-acid side chains on one side of the sliding interface (Figs. S2 and S3).

Side-chain conformations are listed in Table 5 ▸. At 215 K, molecule *B* is disordered over two *gauche* positions (Fig. 2 ▸ *b*), with *gauche*+ as the most populated conformation (Table 5 ▸). Upon cooling to 190 K its occupancy is reduced from 0.602 (7) to 0.100 (6) (Fig. S4), and at 110 K only *gauche*− remains (Fig. 2 ▸ *a*). It follows from the previous data (Görbitz, 2010 ▸) that this ordering upon cooling occurs regardless of whether the phase transition 1 takes place or not (see above).

As the temperature increases between 209 and 355 K, there is a gradual introduction of disorder for molecules *A*, *B* and *C*, while molecule *D* remains fully ordered even at 330 K (Fig. 2 ▸ *c*). At transition 2 the disorder reaches a state where the distinction between *A* and *B* and between *C* and *D* is lost, leaving just two crystallographically independent molecules at 365 K (Fig. 2 ▸ *d*), each with its own distribution of side-chain rotamers, Table 5 ▸. Symmetry consequently increases as the pseudo-twofold rotation axes of the *P*2_1_ structure at 215 K in Fig. 3 ▸(*b*) become proper symmetry elements of a *C*2 unit cell at 365 K (Fig. 3 ▸ *c*).

### 3.3. l-Nva   

**Figure 5:**
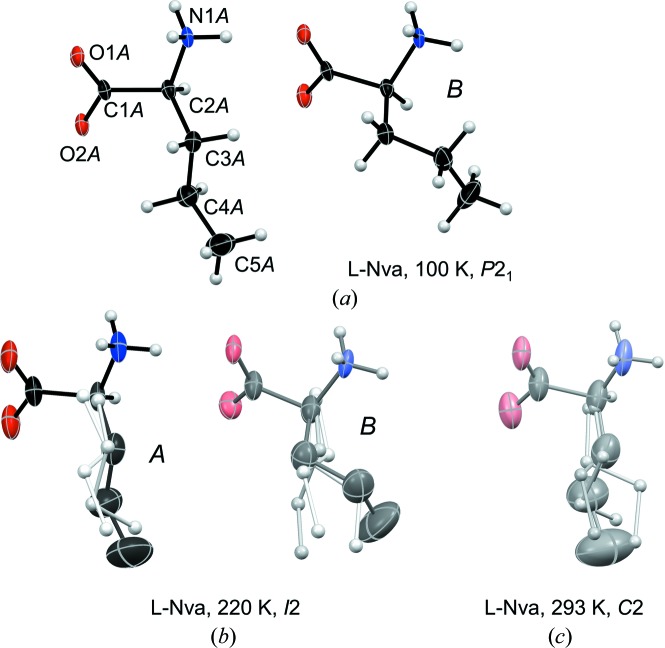
Molecular structure of l-Nva at (*a*) 100, (*b*) 220 and (*c*) 293 K with atomic coloring scheme as in Fig. 2 ▸. Side-chain H atoms have been omitted for disordered structures, and only the most populated conformation is shown in ellipsoid representation; the side chains of minor orientations being shown in ball-and-stick style with spheres of arbitrary size (the polar heads are omitted due to extensive overlap).

The two molecules in the asymmetric unit of l-Nva at 100 K (Fig. 5 ▸ *a*) participate in L2-type hydrogen-bonded sheets (Fig. 4 ▸), not Lx sheets as l-Abu, l-Nle and most other amino acids that do not branch at C_β_ or C_γ_ (Görbitz *et al.*, 2009 ▸). At low temperatures, l-Nva is thus isostructural to its analog with an iso­propyl side chain, l-Val (Dalhus & Görbitz, 1996*a* ▸). There is no side-chain disorder below transition 1 (Fig. S5).

**Figure 6:**
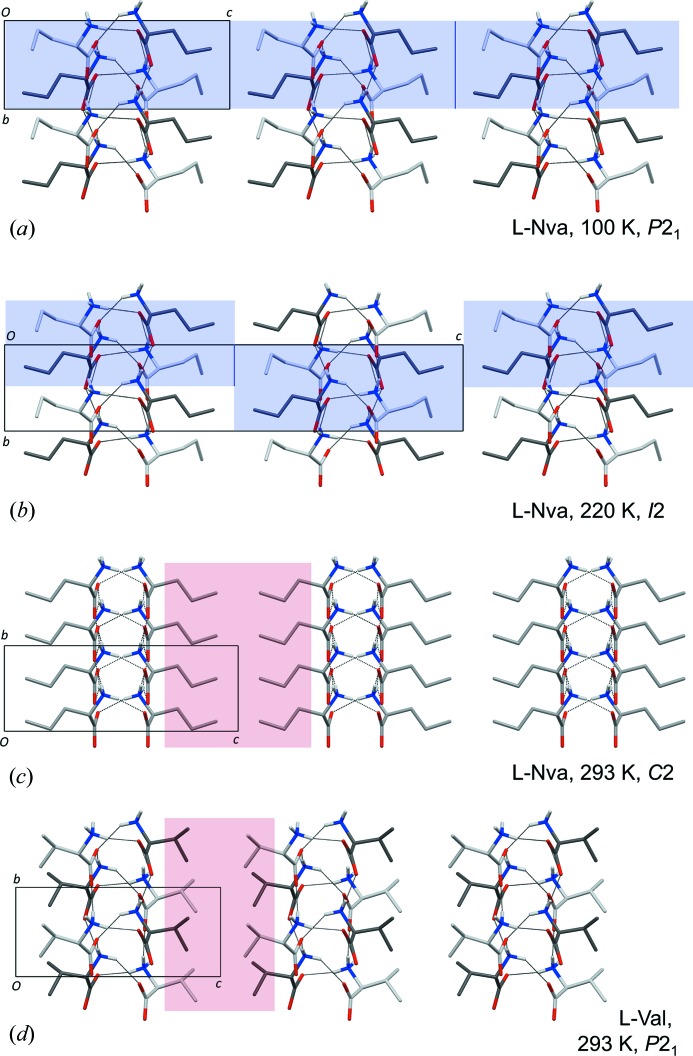
Crystal packing of l-Nva at (*a*) 100 K, (*b*) 220 K and (*c*) 293 K and (*d*) of l-Val at 293 K (Torii & Iitaka, 1970 ▸), all viewed along the *a*-axis. At the two lowest temperatures, C atoms of molecule *A* are colored in dark grey, while those of molecule *B* are light grey. Similar sections of a molecular bilayer of l-Nva are highlighted by blue shading in (*a*) and (*b*) to highlight the sliding half a unit-cell length along the vertical 5.2 Å axis during transition 1. Red shades in (*c*) and (*d*) cover the hydrophobic regions at room temperature, of width 8.82 Å for l-Nva and 6.45 Å for l-Val (calculated as the distance between planes running through the centers of the C_α_—C_β_ bonds).

Upon heating through transition 1 at 207 K (Fig. 1 ▸), sliding of molecular bilayers occurs both along the monoclinic axis (Figs. 6 ▸ *a* and *b*) and the 9.6 Å *a*-axis (Fig. S6). These displacive changes, linked with a shift in space group from *P*2_1_ to *I*2, are equivalent to those observed for the amino acid racemates and quasiracemates described above. Simultaneously, disorder is introduced for both side chains (Fig. 5 ▸ *b*).

**Figure 7:**
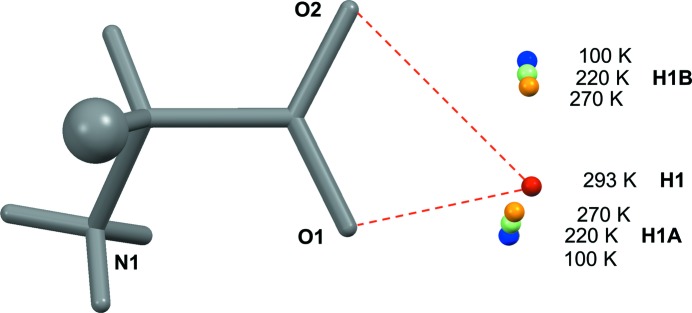
Temperature-dependent positions of H atoms interacting with the *syn* lone pairs of the l-Nva carboxylate group. From their starting positions at 100 K (blue), both H1*B* and H1*A* move towards more central positions as the temperature increases through 220 K (green) to 270 K (orange). After transition 2 in Fig. 1 ▸, the distinction between molecule *A* and *B* (Fig. 5 ▸) has disappeared, giving a single type of three-centered interaction (dashed lines) at 293 K (red).

While all other amino acids investigated so far retain their basic hydrogen-bonding pattern during a phase transition, transition 2 of l-Nva at 273 K (Fig. 1 ▸) concerns a shift from L2–L2 to Lx–Lx with *Z*′ being reduced from 2 (space group *I*2, *Z* = 8) to 1 (*C*2, *Z* = 4, Fig. 5 ▸ *c*). Major shifts in molecular positions upon the change of crystal symmetry are not required, but minor reorientations can be seen by comparing Figs. 6 ▸(*b*) and (*c*). This unprecedented transition appears sharp in DSC, but a plot of the positions of H atoms accepted by the carboxylate *syn* lone pairs (Fig. 7 ▸) makes it evident that H1*A* and H1*B* atoms gradually migrate towards more centered positions between 100 and 270 K (*i.e.* through transition 1). An abrupt final shift results in a single three-centered interaction in an Lx sheet at 293 K (Fig. 4 ▸).

The unit-cell volume and density of l-Nva are in Table 7 ▸ compared with corresponding values for l-Val. The large 15% difference at room temperature is associated with the inefficient stacking of the long l-Nva side chains compared with the isopropyl chain of l-Val (Figs. 6 ▸ *c* and *d*).

Regarding transition 3 in Fig. 1 ▸ at 300 K, the β angle increases from 104.770 (5)° at 293 K to 112.2 (2)° at 320 K (Table S7). As the thermal effect is very weak, any significant change in disorder can be excluded, so this transition is probably associated with minor bilayer sliding and rearrangements of the side chains. As the structure at 293 K is already disordered over four positions, further efforts to resolve this matter were considered futile.

### 3.4. l-Nle   

**Figure 8:**
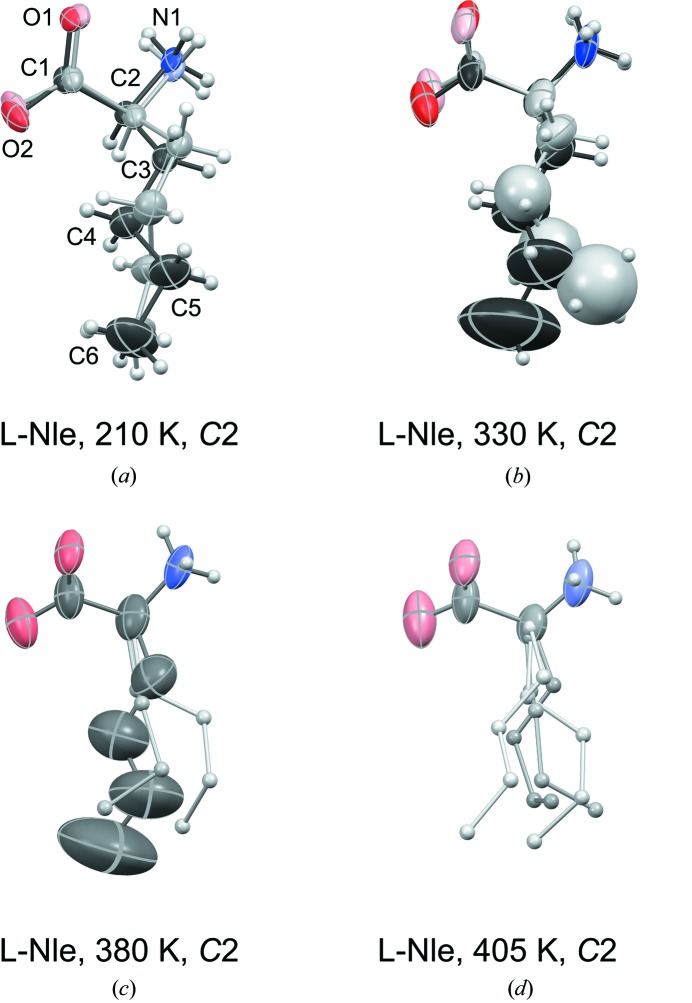
Evolution of the molecular structure of l-Nle upon heating from 210 K in (*a*) to 405 K in (*d*). Style as in Fig. 5 ▸ except that at 405 K all four side-chain conformations are shown in ball-and-stick style with spheres of arbitrary size. Atomic numbering is included for the most populated conformation at 210 K.

**Figure 9:**
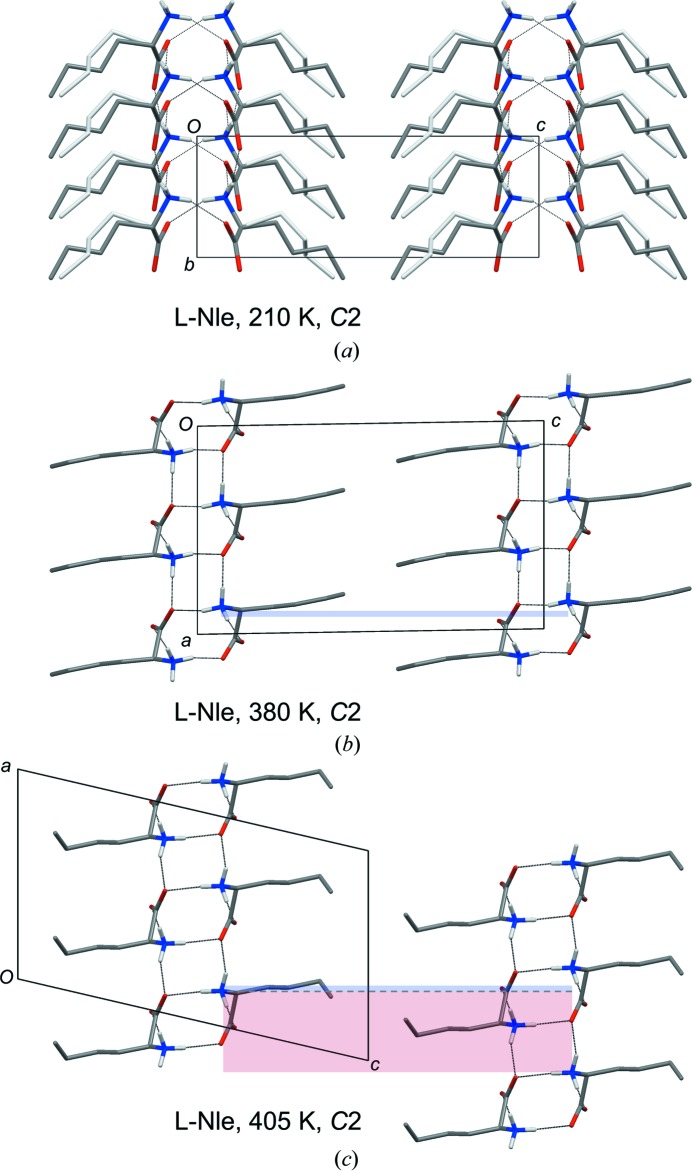
(*a*) Crystal packing of l-Nle at 210 K viewed along the *a*-axis. C atoms of the least populated side-chain conformation are shown in light grey color. (*b*) Crystal packing at 380 K viewed along the *b*-axis. Only the most populated side-chain conformation is included. (*c*) Equivalent to (*b*), but at 405 K. The narrow blue rectangular shade in (*b*) shows the 0.26 Å vertical ‘up’ offset of the right molecule compared with the left as the result of the 90.916 (5)° β angle. In (*c*) the shift is ‘down’ 3.76 Å [red shaded rectangle, β = 103.11 (2)°, note different origin] for a total slide of 4.01 Å along the vertical 9.6 Å *a*-axis.

The room-temperature structure of l-Nle, refined to a reasonable *R*-factor of 0.057, was described by Torii & Iitaka (1973 ▸) as fully ordered, but the non-uniform C—C bond lengths in the side chain, 1.487, 1.546, 1.426 and 1.552 Å (from C_α_ to C_∊_), made us suspect a residual disorder. We confirmed this at an even lower temperature, 210 K, for which refinement to an *R*-factor of 0.036 (Table 3 ▸) revealed one major side-chain orientation of occupancy 0.700 (7), with N1—C2—C3—C4, C2—C3—C4—C5 and C3—C4—C5—C6 torsion angles all *trans*, and one minor orientation of occupancy 0.300 (7), with *trans*, *gauche*+, *trans* torsion angles (Fig. 8 ▸ *a* and Table 5 ▸). Bond lengths in the side chain of the major conformation are now 1.525 (4), 1.530 (6), 1.518 (5) and 1.531 (8) Å from C2 to C6. The crystal packing, with an Lx–Lx hydrogen-bonding pattern (Fig. 9 ▸ *a*), resembles that observed for l-Nva at 293 K (Fig. 6 ▸ *c*) and l-Abu at all temperatures. Due to steric conflict, two neighboring molecules along the *ab*-diagonal cannot both have the minor side-chain conformation.

Using the disordered, but well defined 210 K structure as a reference for l-Nle, increasing temperature brings about conformational rearrangements and increasing disorder, corresponding to the relatively high transition entropy of 6.3 J mol^−1^ K^−1^, Table 6 ▸. This is labelled as transition 3 in Fig. 1 ▸. Numerous ways to refine the side-chain disorder above room temperature were tested, and we settled for two positions at 330 K (Fig. 8 ▸ *b*) and three at 380 K (Fig. 8 ▸ *c*, Table 5 ▸). The space group remains *C*2, but Table 3 ▸ reveals a decrease for the β-angle from 97.628 (2)° at 210 K to 90.916 (5)° at 380 K.

Transition 4 at 395 K in Fig. 1 ▸ concerns a 4.01 Å slide along the 9.6 Å *a*-axis (Figs. 9 ▸ *b* and *c*). The β-angle increases to 103.11 (2)° without changes to the space group or the hydrogen-bonding pattern. Unlike the displacive transition for l-Nva at 207 K described above, no concomitant slide along the *b*-axis is seen (Fig. S7). Sliding along a single axis also occurs for the quasiracemate l-Nle:d-Met (Görbitz & Karen, 2015 ▸), but in a two-step manner.

Our first attempt to solve the 180 K structure was based on a monoclinic *C*2 unit cell with *a* = 28.579 (4), *b* = 5.2472 (8), *c* = 14.759 (2) Å and β = 97.983 (5)°, which can be derived from the unit cell at 210 K (Table 3 ▸) by tripling the length of *a*. As *Z*′ is then increased from 1 to 3, we initially assumed that the 0.700 (7):0.300 (7) disorder between two side-chain conformations at 210 K had become crystallographically ordered 2:1 at 180 K. Subsequent structure refinement was, however, unsatisfactory, with *R* > 0.10 and unreasonable thermal displacement ellipsoids (Fig. S8). A closer look at the diffraction pattern (Fig. S9) immediately revealed something out of the ordinary, two types of reflections being identified: the strong ones indexed on the lattice of the 210 K structure and weak satellites defining a vector **q** = 0.6978**a** ^*^ − 0.1095**c** ^*^ of a modulated structure in the (3 + 1)-dimensional superspace introduced by de Wolff *et al.* (1981 ▸). The new integration of frames was made by *CrysAlis* software (Agilent, 2014 ▸), and the space-group test in *JANA*2006 (Petříček *et al.*, 2014 ▸) proved the superspace group *C*2(α,0,γ)0. With the 210 K structure as a starting model, molecular modulations as well as site occupancies were refined to an almost perfect spatial separation of the two conformers for either of the alternative modulation functions, harmonic or crenel (Table S3). The harmonic model, which needs just three additional parameters, gives a significantly better fit. The result shows that in the 180 K structure it is the ‘order’ of the chains that is incommensurably modulated, yet it is an order as manifested by the relatively high entropy of 2.75 J mol K^−1^ for transition 2 in Fig. 1 ▸.

**Figure 10:**
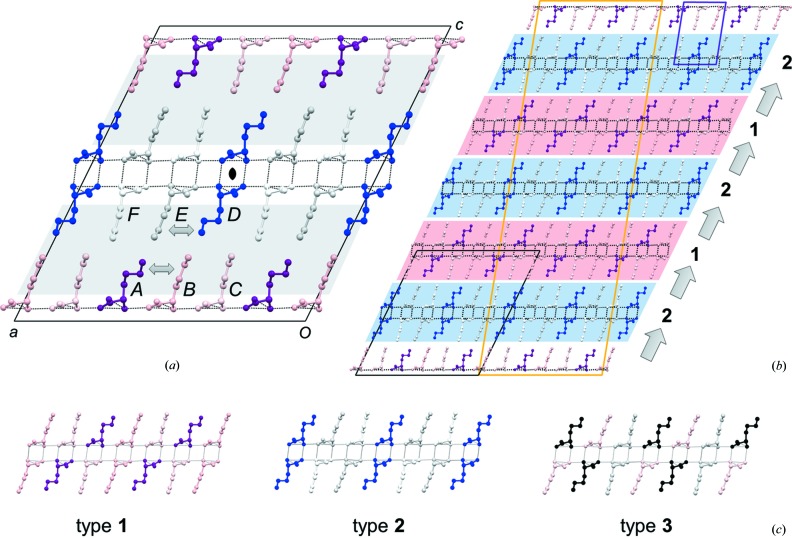
(*a*) The *C*2 unit cell with two bilayers for l-Nle at 100 K. H atoms are omitted, hydrogen bonds are dotted. Molecules *B* (pink), *C* (light pink), *E* (light grey) and *F* (white) have the same side-chain conformation, but *B* and *E* are tilted slightly to relieve close contacts (double arrows) with *A* (purple) and *D* (blue). The two hydrophobic regions in the unit cell (grey shades) are related by the central twofold rotation axis. (*b*) Comparison of the 100 K cell (black edges) with the commensurate supercell (orange) and the unit cell at 210 K (purple). (*c*) The three possible types of bilayers with a 2:1 conformational distribution between *trans*, *trans*, *trans* (light molecules) and *trans*, *gauche*+, *trans* (dark molecules). Type **3** (grey and black molecules) has not been observed experimentally. Red and blue shadings in (*b*) highlight type **1** and type **2** bilayers and the **1**–**2**–**1–2** stacking sequence along the *c*-axis (arrows).

At 100 K yet another structure is obtained. This is also modulated, with vector **q** = 2/3**a** ^*^ − 1/6**c** ^*^, *i.e.* commensurate within experimental accuracy. Hence it has a supercell of six bilayers (Fig. 10 ▸ *b*); a 3 × 1 × 6 multiple of the *C*2 unit cell at 210 K. The parameters of the modulated refinement are listed in the Table S4. The difference in refinement fits for the two modulation functions (harmonic and crenel) is not so pronounced as at 180 K. This means that in this commensurate 100 K phase both conformers are fully separated, as the tiny residual disorder of the incommensurate modulation has been removed (assuming that the same incommensurate phase is obtained on cooling as on heating, as indicated by two additional, partial data sets from a less favorable specimen). A comparison of distances, angles and torsion angles for the 210, 180 and 100 K phases with details of the refinement models is in Table S5.

The solution of the 100 K phase provided an opportunity to study how the mutual molecular interactions affect the geometries of both conformers in their independent positions. The relatively large ratio of independent reflections to the number of refined parameters for the commensurate model with the crenel function (9704/186 ≃ 52) allows a refinement in which the condition of rigidity of molecules is suppressed. To keep the number of refined parameters low, we did constrain the anisotropic thermal-displacement parameters to be the same for the two conformers, and let the atomic coordinates be freely determined by the modulation function. The observed lowering of the *R*-factor indicates that both conformers are slightly but significantly affected by the interactions of their side chains, violating partially the rigid-body model. To model these interactions, the rigid-body condition was applied to three groups of molecules: the molecule pair *A* + *D* (the conformer *trans*, *gauche*+, *trans*) and to the molecule pairs *B* + *E* and *C* + *F* of the other conformer (*trans*, *trans*, *trans*). The result in Table S6 shows that the bond lengths are kept uniform, suggesting that the interactions affect mainly the torsion angles of the conformers.

**Figure 11:**
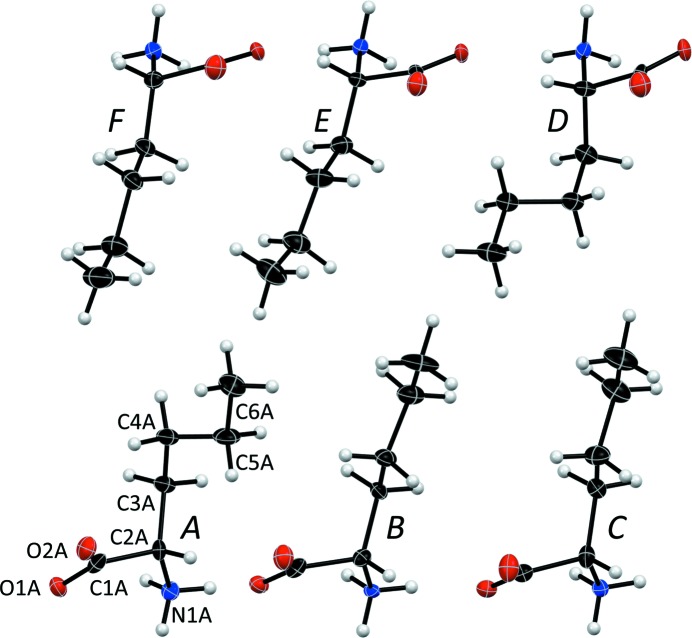
The six molecules *A* to *D* in the asymmetric unit of the *C*2 unit cell of l-Nle at 100 K. Atomic numbers are given for molecule *A*.

The 100 K data set can also be refined on a regular 3 × 1 × 2 unit cell of two bilayers shown in Fig. 10 ▸(*a*). The space group is then *C*2 (Table 3 ▸) with six molecules in the asymmetric unit (Fig. 11 ▸). Further analysis shows that an l-Nle bilayer with a 2:1 distribution between side-chain conformations *trans*,*trans*,*trans* (4 molecules) and *trans*,*gauche*+,*trans* (2 molecules) could occur in three different types **1**, **2** and **3** (Fig. 10 ▸ *c*). The observed stacking sequence along the *c*-axis is **1**–**2**–**1**–**2** (Fig. 10 ▸ *b*), with no trace of type **3**. Only four other amino-acid structures have two types of bilayers in the crystal: The *I*2 and *P*2_1_ phases of l-Abu discussed above (Fig. 3 ▸ *a* and 3*b*) as well as form (I) (Ihlefeldt *et al.*, 2014 ▸) and form (III) (Mossou *et al.*, 2014 ▸) of l-phenylalanine. These four structures have in common not only a *Z*′ value of 4, but also that adjacent bilayers are antiparallel. All other structures have identical, parallel bilayers. Considering that hydrogen-bonding patterns are unaffected by the side-chain conformations, it is indeed quite remarkable that l-Nle forms a modulated, but ordered crystalline **1**–**2**–**1**–**2** arrangement at 100 K instead of any of the simpler **1**–**1**–**1**, **2**–**2**–**2** or **3**–**3**–**3** alternatives.

Transition 1 is not seen in the DSC scan on cooling, but visible on heating (Fig. 1 ▸). Our 180 K data set was collected after first cooling the structure to 100 K, but subsequent testing (with somewhat lower quality crystals) confirmed that both modulation and the apparent *C*2 unit cell with *Z*′ = 3 is observed at 180 K both on cooling and on heating, so the reason why we do not observe the weak transition 1 upon cooling is not clear. The details of the transitions to and between the modulated l-Nle phases will be subject to future, more comprehensive investigations, which will also include monitoring obvious crystal-to-crystal variation.

### 3.5. l-Met   

**Figure 12:**
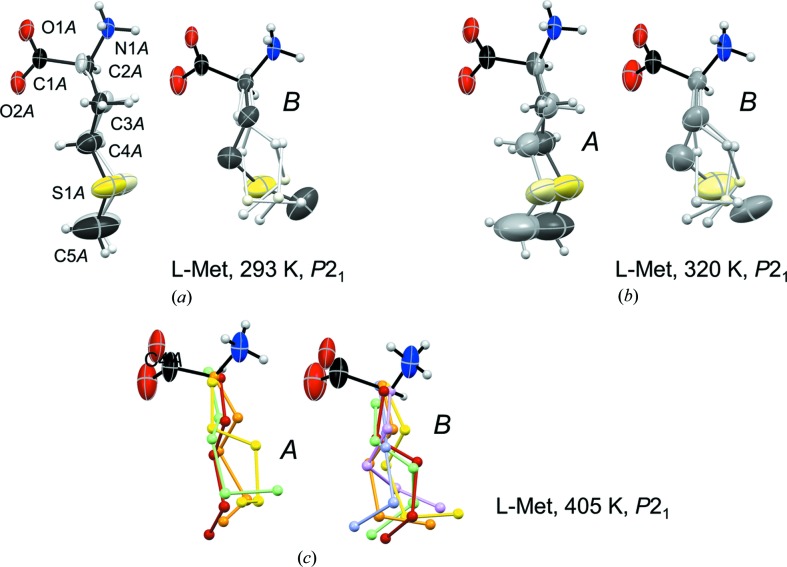
Molecular structures of l-Met at (*a*) 293 K, (*b*) 320 K and (*c*) 405 K; style as in Figs. 5 ▸ and 8 ▸ except that at 405 K side-chain conformations are colored in a rainbow sequence (red, orange, yellow, light green, light blue, light violet) from highest to lowest occupancy. Only a single set of coordinates was refined for O1, O2, N1 and C1 of each amino acid; these atoms thus occur in regular red, blue and black colors.

No side-chain disorder was described for l-Met at 120 K (Dalhus & Görbitz, 1996*a* ▸), 150 K (Sadler *et al.*, 2005 ▸) or at room temperature (Torii & Iitaka, 1973 ▸). The present structure refinement reveals, on the other hand, two side-chain conformations for molecule *A* and four for molecule *B* at 293 K (Fig. 12 ▸ *a*). The two positions for the molecule *A* side chain both define the same formal *trans*, *trans*, *trans* orientation for N1*A*–C2*A*–C3*A*–C4*A*, C2*A*–C3*A*–C4*A*–S1*A*, C3*A*–C4*A*–S1*A*–C5, with small separations between the two alternative positions for each atom. Considering that short intermolecular distances (less than sum of van der Waals radii) to atoms in the side chain of molecule *B* should be avoided, the major orientation of *A* [occupancy 0.830 (4)] is associated with the *trans*, *gauche*+, *gauche*+ and *trans*, *trans*, *gauche*− conformations for *B* [combined occupancy 0.809 (3)]. Conversely, the *A* side chain adopts the minor orientation when the side chain of *B* is either *gauche*−, *trans*, *gauche*+ or *gauche*−, *gauche*−, *gauche*−, Table 5 ▸.

**Figure 13:**
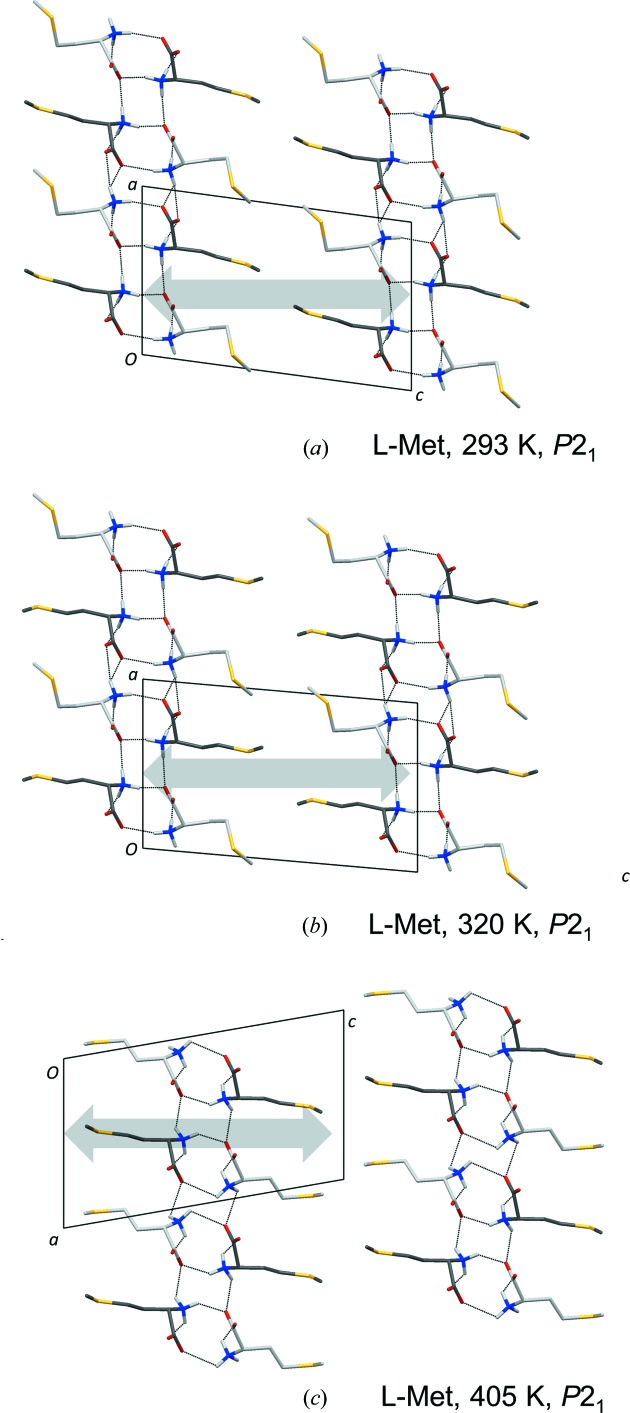
Crystal packing of l-Met at (*a*) 293 K, (*b*) 320 K and (*c*) 405 K viewed along the *b*-axis with C atoms of molecule *A* colored in dark grey and those of molecule *B* in light grey. As in previous packing diagrams, only the most populated side-chain conformation is shown for each molecule. The length of the gray double arrow at 293 K represents the distance between the centers of neighboring molecular bilayers [calculated as *c*·sin(β)]. Arrows of the same length at 320 and 405 K illustrate the expansion perpendicular to the molecular bilayers as a function of temperature and phase transitions.

Fig. 1 ▸ shows two previously unknown transitions for l-Met above room temperature. Upon heating from room temperature only minor structural changes are apparent above transition 1, despite a rather high value for Δ*S* of 4.66 J mol^−1^ K^−1^, Table 6 ▸. These are associated with increased populations for the minor side-chain conformations (Fig. 12 ▸ *b* and Table 5 ▸) as well as a smaller value for the β angle and increased interlayer spacing (Table 4 ▸ and Figs. 13 ▸ *a* and 13*b*). By comparison, structural changes between the 320 and 405 K forms are much more evident, even though the *P*2_1_ space group is retained. Molecular disorder is very complex for l-Met above transition 2; the high number of electrons for the side-chain sulfur atom allowing detection of atomic positions with low occupancy. The final refinement model displayed in Fig. 12 ▸(*c*) employed no less than four different side-chain positions for molecule *A* and six for molecule *B*, but the true number of conformations in the crystal may actually be even larger. More elaborate refinement models did not, however, result in a significant reduction of the *R*-factor. In addition to the development of multifold disorder above transition 2, there is also a slide along the 9.5 Å axis (Fig. 13 ▸ *c*) that closely mimics the slide at transition 4 of l-Nle taking place in the same temperature range. Sliding along the *b*-axis is similarly absent (Fig. S11).

Cell parameters recorded at 430 K (Table S7) deviate primarily from those obtained at 405 K in terms of a smaller β angle, 94.13 (10)° *versus* 99.869 (13)°, and what appears to be a reduction in unit-cell volume from 791.3 (5) to 773 (7) Å^3^. Due to the complex disorder at 405 K, no full data collection was carried out to further investigate this matter.

It is noteworthy that the hydrogen-bonding patterns of l-Nle and l-Met are different at all temperatures, Lx–Lx *versus* L2–L2. The change triggered by the associated—C^δ^H_2_— to —S— substitution suggests, like the temperature-induced transition 2 for l-Nva discussed above, that these patterns have quite similar energies. This is furthermore substantiated by l-Abu (Görbitz, 2010 ▸) and the monoclinic polymorph of l-Cys (Görbitz & Dalhus, 1996 ▸), for which the related CH_3_-to-SH substitution induces a shift from Lx–Lx to L2–L2 hydrogen bonding.

## 4. Conclusions   

Prior to the present investigation, only a single temperature-induced phase transition was known for an enantiomeric amino acid, pertaining to orthorhombic l-Cys (Moggach *et al.*, 2005 ▸), where a disordered thiol group becomes ordered at 30 K. This picture is changed dramatically by the results presented here for l-methionine (l-Met), l-aminobutyric acid (l-Abu), l-norvaline (l-Nva) and l-norleucine (l-Nle). Together, these four amino acids with linear, hydrophobic side chains display at least 12 phase transitions, which are associated with a surprising variety of physical processes in the crystal. These include concerted side-chain rearrangements (l-Abu), regular symmetry increase due to developing disorder (l-Abu), simultaneous sliding along two crystallographic axes (l-Nva), hydrogen-bond pattern rearrangement (l-Nva), more gradual introduction of complex disorder or change in rotamer composition (l-Nle, l-Met), displacive transition along a single axis (l-Nle, l-Met) and finally transitions to commensurate and incommensurately modulated structures (l-Nle), the first of their kind for amino acids. For the amino acid counterparts with branched side chains, such as l-Val, l-Ile and l-Leu, no comparable transitions have been found, highlighting the remarkable properties of the four compounds studied here.

## Supplementary Material

Crystal structure: contains datablock(s) Abu190, Abu215, Abu330, Abu365, Nva100, Nva190, Nva220, Nva270, Nva293, Nle100super, Nle210, Nle330, Nle380, Nle405, Met293, Met320, Met405. DOI: 10.1107/S2052252516010472/ed5010sup1.cif

Structure factors: contains datablock(s) Abu190. DOI: 10.1107/S2052252516010472/ed5010Abu190sup2.hkl

Structure factors: contains datablock(s) Abu215. DOI: 10.1107/S2052252516010472/ed5010Abu215sup3.hkl

Structure factors: contains datablock(s) Abu330. DOI: 10.1107/S2052252516010472/ed5010Abu330sup4.hkl

Structure factors: contains datablock(s) Abu365. DOI: 10.1107/S2052252516010472/ed5010Abu365sup5.hkl

Structure factors: contains datablock(s) Nva100. DOI: 10.1107/S2052252516010472/ed5010Nva100sup6.hkl

Structure factors: contains datablock(s) Nva190. DOI: 10.1107/S2052252516010472/ed5010Nva190sup7.hkl

Structure factors: contains datablock(s) Nva220. DOI: 10.1107/S2052252516010472/ed5010Nva220sup8.hkl

Structure factors: contains datablock(s) Nva270. DOI: 10.1107/S2052252516010472/ed5010Nva270sup9.hkl

Structure factors: contains datablock(s) Nva293. DOI: 10.1107/S2052252516010472/ed5010Nva293sup10.hkl

Structure factors: contains datablock(s) Nle100super. DOI: 10.1107/S2052252516010472/ed5010Nle100supersup11.hkl

Structure factors: contains datablock(s) Nle210. DOI: 10.1107/S2052252516010472/ed5010Nle210sup12.hkl

Structure factors: contains datablock(s) Nle330. DOI: 10.1107/S2052252516010472/ed5010Nle330sup13.hkl

Structure factors: contains datablock(s) Nle380. DOI: 10.1107/S2052252516010472/ed5010Nle380sup14.hkl

Structure factors: contains datablock(s) Nle405. DOI: 10.1107/S2052252516010472/ed5010Nle405sup15.hkl

Structure factors: contains datablock(s) Met293. DOI: 10.1107/S2052252516010472/ed5010Met293sup16.hkl

Structure factors: contains datablock(s) Met320. DOI: 10.1107/S2052252516010472/ed5010Met320sup17.hkl

Structure factors: contains datablock(s) Met405. DOI: 10.1107/S2052252516010472/ed5010Met405sup18.hkl

Supporting figures and tables. DOI: 10.1107/S2052252516010472/ed5010sup19.pdf

CCDC references: 1472679, 1472680, 1472681, 1472682, 1472683, 1472684, 1472685, 1472686, 1472687, 1472688, 1472689, 1472690, 1472691, 1472692, 1472693, 1472694, 1472695

## Figures and Tables

**Table 1:** Crystal data for L-Abu (C_4_H_9_NO_2_)

Temperature (K)	110†	110†	190	215	330	365
Space group	*I*2	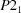	*I*2	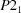	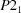	*C*2
*a* (Å)	9.646 (2)	9.614 (6)	9.6246 (7)	9.6176 (9)	9.6132 (6)	9.6233 (12)
*b* (Å)	5.2145 (12)	5.227 (3)	5.2079 (4)	5.2126 (5)	5.2239 (3)	5.2274 (6)
*c* (Å)	42.885 (10)	21.385 (13)	43.103 (3)	21.768 (2)	22.4134 (16)	22.877 (3)
β (°)	100.295 (3)	100.326 (7)	100.201 (2)	101.123 (3)	101.453 (2)	100.764 (4)
*V* (Å^3^)	2122.5 (2)	1057.1 (11)	2126.4 (3)	1070.80 (17)	1103.15 (12)	1130.6 (2)
*Z*, *Z*′	16, 4	8, 4	16, 4	8, 4	8, 4	8, 2
*N*_measured_	–	–	14 739	13 667	11 282	3209
*N*_unique_	–	–	5251	3914	3841	1787
*N*_observed_ [*F* ^2^ > 2σ(*F* ^2^)]	–	–	4591	3293	2727	1322
*n*_par_	–	–	266	292	336	221
*R*_int_	–	–	0.040	0.032	0.155	0.035
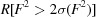	–	–	0.039	0.038	0.077	0.047
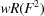	–	–	0.105	0.092	0.189	0.103
*S*	–	–	1.065	1.075	1.082	1.167
CCDC#	775214	775213	1472679	1472688	1472689	1472690

† Görbitz (2010 ▸); CSD refcodes HUWSOI01 (*I*2) and HUWSOI (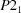).

**Table 2:** Crystal data for L-Nva (C_5_H_11_NO_2_)

Temperature (K)	100	190	220	270	293
Space group	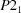	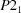	*I*2	*I*2	*C*2
*a* (Å)	9.6123 (15)	9.604 (3)	9.5868 (8)	9.5855 (15)	9.589 (4)
*b* (Å)	5.1222 (9)	5.1222 (16)	5.1560 (4)	5.1752 (9)	5.2054 (19)
*c* (Å)	13.183 (2)	13.352 (4)	27.477 (1)	27.959 (5)	14.698 (7)
β (°)	98.609 (5)	97.137 (7)	93.203 (3)	92.057 (3)	104.770 (5)
*V* (Å^3^)	641.78 (19)	651.7 (4)	1356.1 (2)	1386.1 (4)	709.4 (5)
*Z*, *Z*′	4, 2	4, 2	8, 2	8, 2	4, 1
*N*_measured_	5169	4679	2100	6794	1748
*N*_unique_	2352	2424	1455	2397	1120
*N*_observed_ [*F* ^2^ > 2σ(*F* ^2^)]	1990	1699	1204	1660	904
*n*_par_	147	147	259	259	156
*R*_int_	0.041	0.051	0.018	0.057	0.016
*R*[*F* ^2^ > 2σ(*F* ^2^)]	0.051	0.052	0.044	0.052	0.039
*wR*(*F* ^2^)	0.106	0.117	0.110	0.135	0.115
CCDC#	1472691	1472692	1472693	1472694	1472695

**Table 3:** Crystal data for L-Nle (C_6_H_13_NO_2_)

Temperature (K)	100†	210	295‡	330	380	405
Space group	*C*2	*C*2	*C*2	*C*2	*C*2	*C*2
*a* (Å)	28.516 (5)	9.5327 (7)	9.550 (5)	9.5633 (15)	9.6033 (15)	9.648 (9)
*b* (Å)	5.2346 (10)	5.2545 (4)	5.260 (5)	5.2287 (9)	5.2223 (8)	5.252 (5)
*c* (Å)	32.233 (6)	14.959 (1)	15.377 (5)	15.674 (3)	15.983 (3)	16.561 (16)
β (°)	116.25 (8)	97.628 (2)	95.60 (5)	93.695 (5)	90.916 (5)	103.11 (2)
*V* (Å^3^)	12994.1 (16)	742.66 (9)	768.75 (3)	782.1 (2)	801.5 (2)	817.2 (13)
*Z*, *Z*′	18, 18	4, 1	4, 1	4, 1	4, 1	4, 1
*N*_measured_	33 113	6243	–	4947	3890	1901
*N*_unique_	8552	1420	–	1389	1412	1283
*N*_observed_ [*F* ^2^ > 2σ(*F* ^2^)]	3859	1217	–	929	708	533
*n*_par_	493	129	–	114	146	115
*R*_int_	0.149	0.027	–	0.039	0.052	0.082
*R*[*F* ^2^ > 2σ(*F* ^2^)]	0.081	0.036	–	0.051	0.071	0.097
*wR*(*F* ^2^)	0.219	0.079	–	0.129	0.189	0.205
CCDC#	1472680	1472681	1207985	1472682	1472683	1472684

† Refinement based on *a*. *hkl* file transformed from the supercell refinement.

‡ Torii & Iitaka (1973 ▸); CSD refcode LNLEUC10.

**Table 4:** Crystal data for L-Met (C_5_H_11_NO_2_S)

Temperature (K)	120†	150‡	293	320	405
Space group	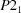	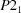	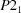	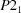	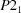
*a* (Å)	9.493 (2)	9.512 (1)	9.5118 (6)	9.5473 (6)	9.588 (4)
*b* (Å)	5.201 (2)	5.209 (1)	5.1936 (4)	5.1830 (3)	5.203 (2)
*c* (Å)	14.831 (3)	14.858 (1)	15.3419 (10)	15.5830 (10)	16.101 (7)
β (°)	99.84 (2)	99.70 (1)	97.635 (2)	94.980 (2)	99.869 (13)
*V* (Å^3^)	721.5 (3)	725.69	751.18 (9)	768.19 (8)	791.3 (5)
*Z*, *Z*′	4, 2	4, 2	4, 2	4, 2	4, 2
*N*_measured_	–	–	15 609	7664	9797
*N*_unique_	–	–	3308	2936	2638
*N*_observed_ [*F* ^2^ > 2σ(*F* ^2^)]	–	–	2682	2082	1642
*n*_par_	–	–	231	248	279
*R*_int_	–	–	0.038	0.046	0.057
*R*[*F* ^2^ > 2σ(*F* ^2^)]	–	–	0.047	0.066	0.117
*wR*(*F* ^2^)	–	–	0.118	0.189	0.293
CCDC#	–	276855	1472685	1472686	1472687

† Dalhus & Görbtiz (1996*a* ▸); CSD refcode LMETON02.

‡ Sadler *et al.* (2005 ▸); CSD refcode LMETON11.

**Table:** The torsion angles listed are N1—C2—C3—C4 (all), C2—C3—C4—C5 (Nva and Nle) or C2—C3—C4—S1 (Met), C3—C4—C5—C6 (Nle) or C3—C4—S1—C5 (Met). *t* = *trans*, 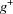 = *gauche* 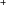, 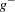 = *gauche* 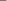.

L-Abu	110 (Görbitz, 2010 ▸)	190	215	330	365
Molecule *A*					
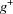	0.000	0.000	0.000	0.23 (2)	0.563 (12)
*t*	1.000	1.000	1.000	0.77 (2)	0.260 (12)
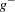	0.000	0.000	0.000	0.000	0.169 (11)
					
Molecule *B*					
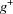	0.000	0.100 (6)	0.602 (7)	0.716 (13)	0.337 (11)
*t*	0.000	0.000	0.000	0.000	0.573 (13)
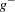	1.000	0.900 (6)	0.398 (7)	0.284 (13)	0.098 (10)
					
Molecule *C*					
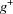	1.000	1.000	1.000	0.69 (2)	–
*t*	0.000	0.000	0.000	0.31 (2)	–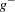	0.000	0.000	0.000	0.000	–
					
Molecule *D*					
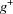	0.000	0.000	0.000	0.000	–
*t*	1.000	1.000	1.000	1.000	–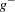	0.000	0.000	0.000	0.000	–

**Table:** 

L-Nva	100 and 190	220	270	293
Molecule *A*				
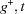	0.000	0.068 (3)	0.1174 (4)	0.250 (9)
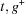	0.000	0.131 (3)	0.1770 (4)	0.197 (13)
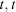	1.000	0.800 (3)	0.7056 (4)	0.369 (14)
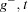	0.000	0.000	0.000	0.184 (9)
				
Molecule *B*				
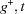	0.000	0.116 (3)	0.1758 (4)	–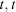	0.000	0.232 (3)	0.3058 (4)	–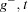	0.000	0.124 (3)	0.1301 (4)	–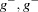	1.000	0.528 (3)	0.3884 (4)	–

**Table:** 

L-Nle	210	330	380	405
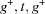	0.000	0.000	0.000	0.287 (14)
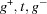	0.000	0.000	0.275 (13)	0.000
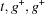	0.000	0.215 (13)	0.000	0.000
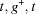	0.300 (7)	0.000	0.000	0.000
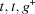	0.000	0.000	0.000	0.367 (15)
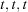	0.700 (7)	0.785 (13)	0.527 (13)	0.156 (14)
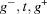	0.000	0.000	0.198 (12)	0.190 (12)

**Table:** 

L-Met	120 and 150	293	320	405
Molecule *A*				
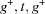	0.000	0.000	0.000	0.093 (13)
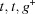	0.000	0.000	0.000	0.28 (2)
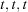	1.000	1.000†	1.000‡	0.50 (2)
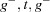	0.000	0.000	0.000	0.130 (13)
				
Molecule *B*				
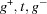	0.000	0.000	0.000	0.139 (12)
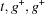	1.000	0.694 (3)	0.406 (2)	0.098 (12)
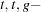	0.000	0.115 (2)	0.148 (2)	0.40 (2)§
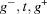	0.000	0.076 (2)	0.203 (2)	0.239 (13)
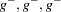	0.000	0.114 (3)	0.241 (2)	0.153 (15)

† Two separate positions, 0.830 (4) and 0.170 (4).

‡ Two separate positions, 0.313 (6) and 0.687 (6).

§ Two separate positions, 0.174 (19) and 0.224 (17).

**Table 6:** Characterization of the transitions by DSC

Transition	Thermal barycenter (K)†	Peak-top temperature (K)†	Hysteresis (K)	Δ*S* (J mol^−1^ K^−1^)	Shape
L-Abu **1**	210	207	6	1.58	Sharp
L-Abu **2**	341	355	0	3.09	Broad
L-Nva **1**	225	207	0	7.2	Very broad
L-Nva **2**	274	273	1	0.21	Sharp
L-Nva **3**	298	300	0	0.08	Broad
L-Nle **1**	171	168	–‡	0.19	Sharp
L-Nle **2**	191	199	0	2.56	Intermediate
L-Nle **3**	323	337	2	6.3	Broad
L-Nle **4**	391	396.5	4	0.3	Sharp
L-Met **1**	300	309	2	4.66	Broad
L-Met **2**	397	395	4	0.38	Intermediate
L-Met **3**	424	424	0	0.10	Intermediate

† Upon heating at 20 K min^−1^.

‡ Extrapolated to zero rate for the temperature change.

§ Observed only upon heating.

**Table 7:** Comparison between L-Val and L-Nva at low and ambient temperatures

Compound	*T* (K)	Space group	*V* (Å^3^)	Change (%)	*D*_*x*_ (g cm^−3^)
L-Val†	120	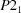	606.0 (1)	–	1.284
L-Nva	100	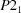	641.78 (19)	+5.9	1.212
L-Val‡	293	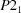	617.1§	–	1.261
L-Nva	293	*C*2	709.4 (5)	+15.0	1.097

† Dalhus & Görbitz (1996*a* ▸).

‡ Torii & Iitaka (1970 ▸).

§ No s.u.s given.
