# Supplementary material for: An exceptional series of phase transitions in hydrophobic amino acids with linear side chains
Source: IUCrJ. 2016 Aug 9;3(Pt 5):341–53. doi: 10.1107/S2052252516010472 (PMC5391856; doi:10.1107/S2052252516010472)
Supplement: Supplementary file 19 [file m-03-00341-sup19.pdf]

IUCrJ

Volume 3 (2016)

Supporting information for article:

An exceptional series of phase transitions in hydrophobic amino acids with linear side chains

Carl Henrik Görbitz, Pavel Karen, Michal Dušek and Vaclav Petrícek

Supporting information **page**

Figure 1S. Schematic phase diagram for L-Abu, L-Nva, L-Nle and L-Met 2

Figure 2S. Model for the L-Abu phase transition 1 3

Figure 3S. Overlay of L-Abu structures at 190 and 215 K 4

Figure 4S. Asymmetric unit of L-Abu at 190 K 5

Figure 5S. Molecular structure of L-Nva at 190 and 270 K 5

Figure 6S. Crystal packing of L-Nva viewed along the *b* axis at 100 and 220 K 6

Figure 7S. Crystal packing of L-Nle viewed along the *a*-axis at 380 and 405 K. 7

Figure 8S. Initial asymmetric unit and crystal packing of L-Nle at 180 K. 8

Figure 9S. Reciprocal lattice of L-Nle at 180 K. 9

Figure 10S. Two selected sections of the L-Nle structure at 180 K. 11

Figure 11S. Crystal packing of L-Met viewed along the *a*-axis at 293, 320 and 405 K. 12

Table 1S. Side-chain torsion angles 13

Table 2S. Hydrogen-bond parameters 15

Table 3S. Experimental and refinement details – L-Nle 180K 18

Table 4S. Experimental and refinement details – L-Nle 100K 19

Table 5S. Molecular geometry (Å, °) of the two L-Nle conformations at 210, 180 and 100 K. 20

Table 6S. Molecular geometry (Å, °) of the two L-Nle conformations at 100 K for three refinement models 21

Table 7S. Unit cell parameters for L-Nva at 320 K and L-Met at 430 K 22

Data transformation for L-Nle at 100 K 23

On hydrogen bonding in the crystal structures of hydrophobic amino acids in their enantiomeric form (including Figures 10S to 12S) 24

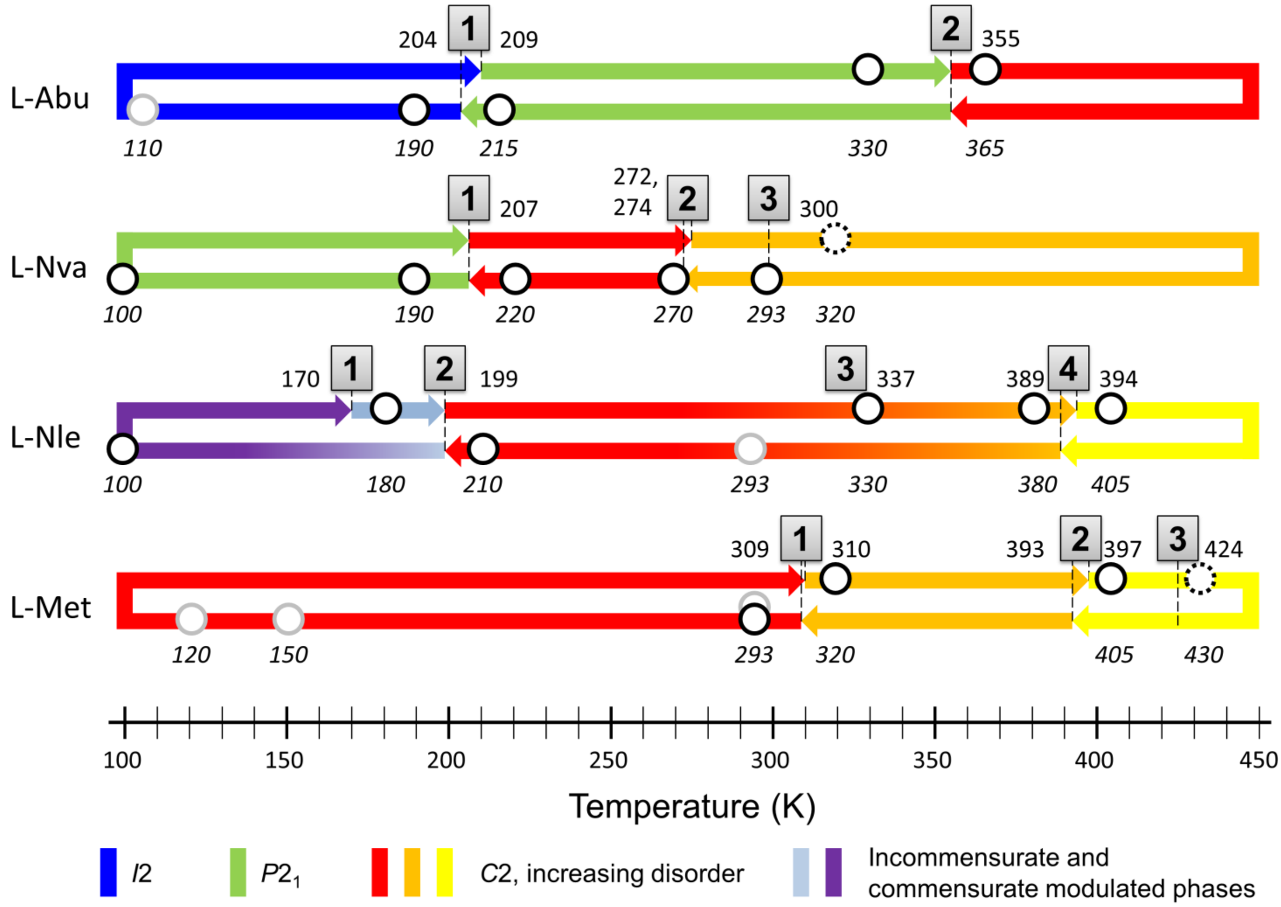

Figure S1 Phase transitions (labelled in grey boxes) for enantiomeric amino acids with linear, hydrophobic side chains on cooling (left arrows) and heating (right arrows); space group color legend is at the bottom. Transitions temperatures appear above each graph; when only one temperature is given hysteresis is 0 K. Circles in black indicate single crystal structure determinations carried out here at temperatures given in italic type face. Grey outline is used for previous investigations for L-Abu, (Görbitz, 2010), L-Nle (Torii & Iitaka, 1973) and L-Met (Torii & Iitaka, 1973, Sadler *et al.*, 2005, Dalhus & Görbitz, 1996a). Dashed circles indicate that only unit cell parameters were determined.

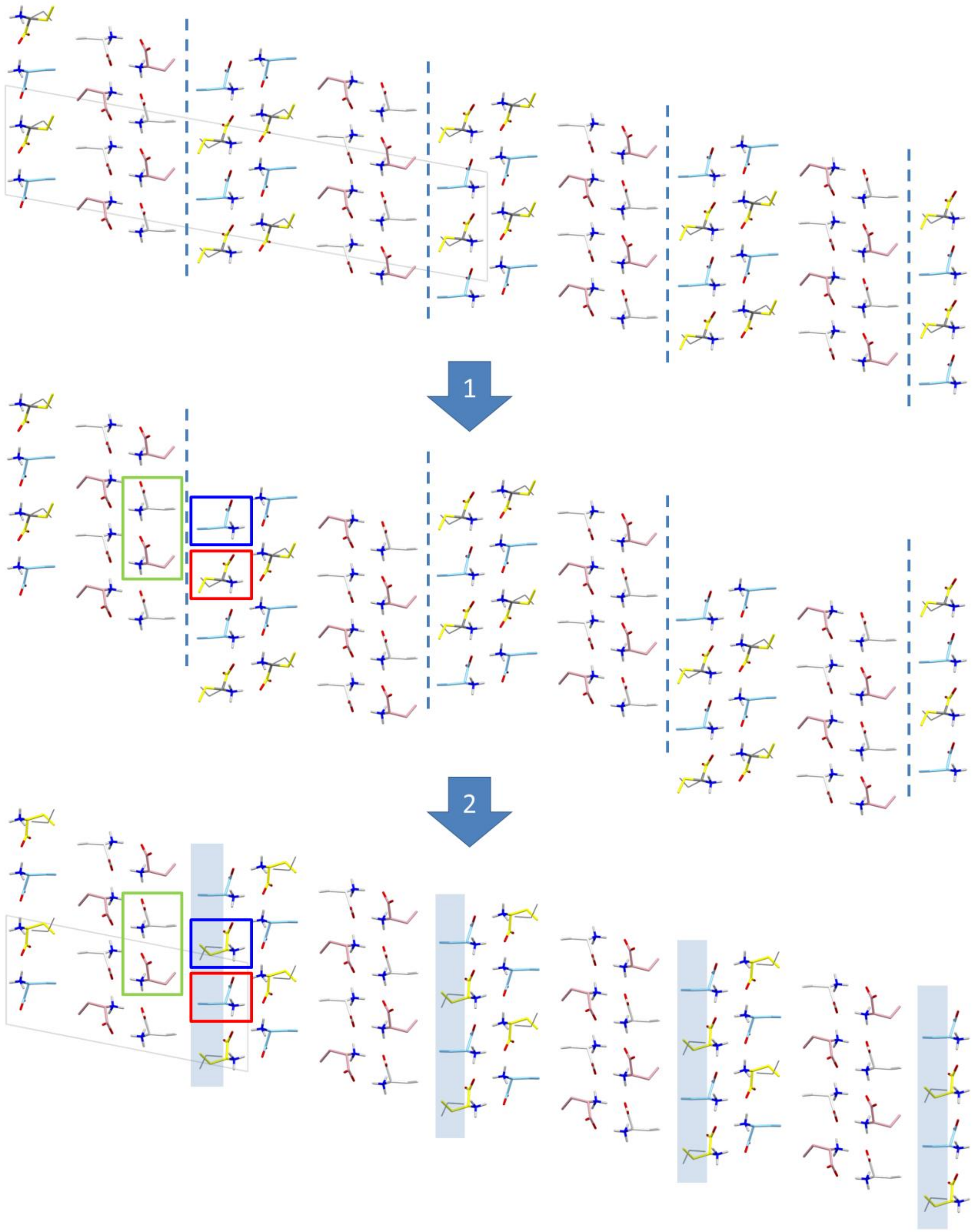

Figure S2 Model for the phase transition 1 between the 190 K *I*2 form of L-Abu (top) and the 215 K $P2_1$ form (bottom). Carbon atoms of molecule *A*, *B*, *C* and *D* are colored in sky blue, yellow, pink and white, respectively The transition probably takes place as one single, concerted process, but is, for simplicity, shown here as two separate steps. In step 1 there is sliding along every second interface between molecular bilayers (dashed lines). In step 2 side-chains in highlighted boxes undergo conformational changes that interchange the conformations of the *A*- and *B*-molecules. Upon heating (as shown from top to bottom), *A*-molecules are then converted from a *trans* conformation to two *gauche* conformations (Table 5) and becomes *B*-molecules (blue boxes), while the opposite takes place for the *B*-molecules (red boxes). On cooling the process is reversed. *C*- and *D*-molecules on the opposite side of the interface (larger, green box) are unaffected.

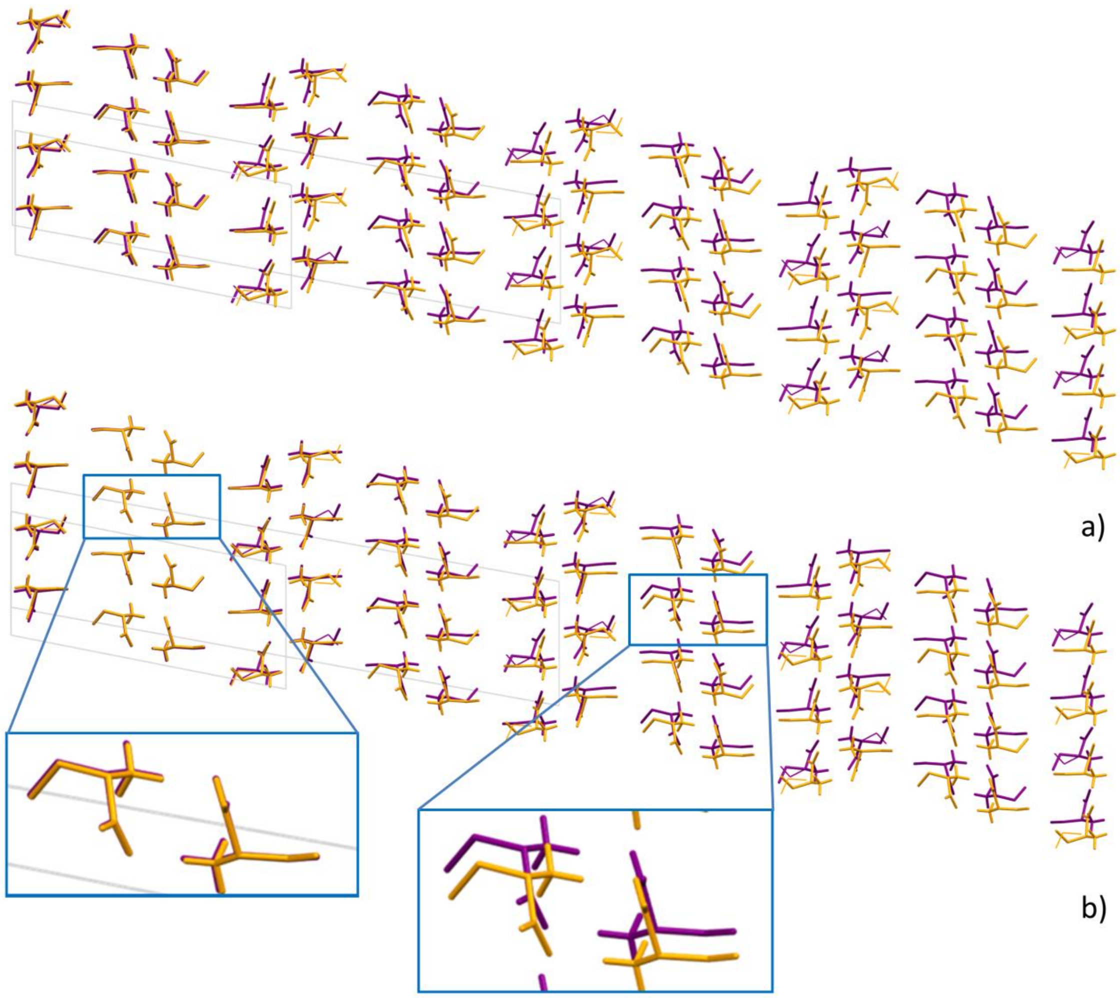

Figure S3 a) Overlay of the crystal packing of L-Abu at 190 K (purple) and 215 K (orange) on the same scale. b) Same overlay, but with the unit cell at 215 K reduced in size by 0.4 % to compensate for thermal expansion. As the sliding of molecular bilayers described in Figure 1S is not exactly half a unit cell length, *C* and *D* molecules that fall on top of each other in the left-hand box, but are slightly displaced in the right-hand box. The β-angle is 100.201(2)° for the large *I*2 unit cell at 190 K and 101.123(3)° for the smaller $P2_1$ unit cell at 215 K.

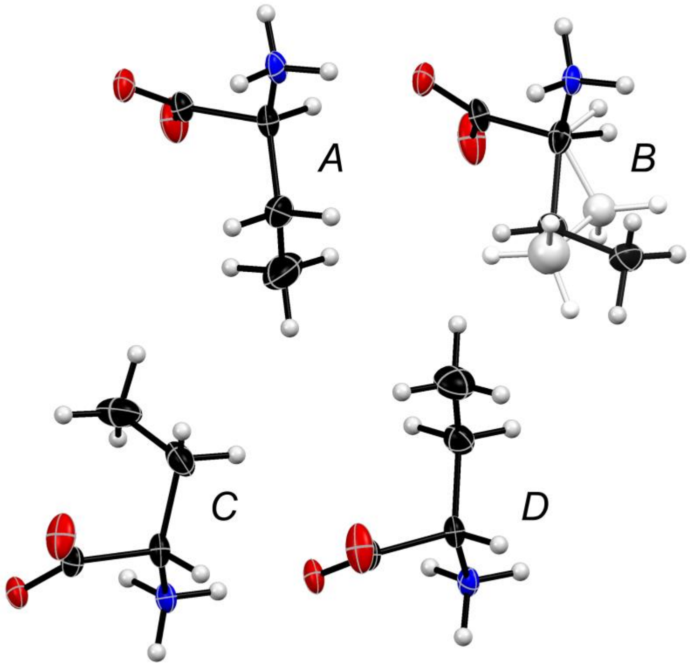

Figure S4 Molecular structure of L-Abu at 190 K. Individual molecules in the asymmetric unit are labelled in italics (*A*, *B*, …). Thermal displacement ellipsoids are shown at the 50 % probability level. The minor conformation for molecule *B*, drawn in lighter color, has occupancy 0.100(6).

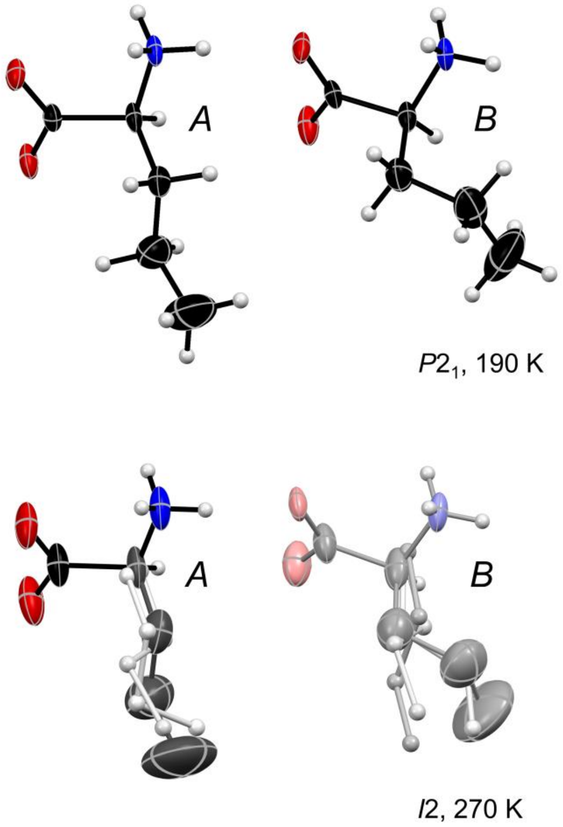

Figure S5 Molecular structure of L-Nva at 190 K and at 270 K. At 270 K side-chain H atoms are omitted, while color depths reflect the occupancy of each conformation as listed in Table 5. Ball-and-stick style with spheres of arbitrary size is used for the minor orientations, their polar heads being omitted due to extensive overlap.

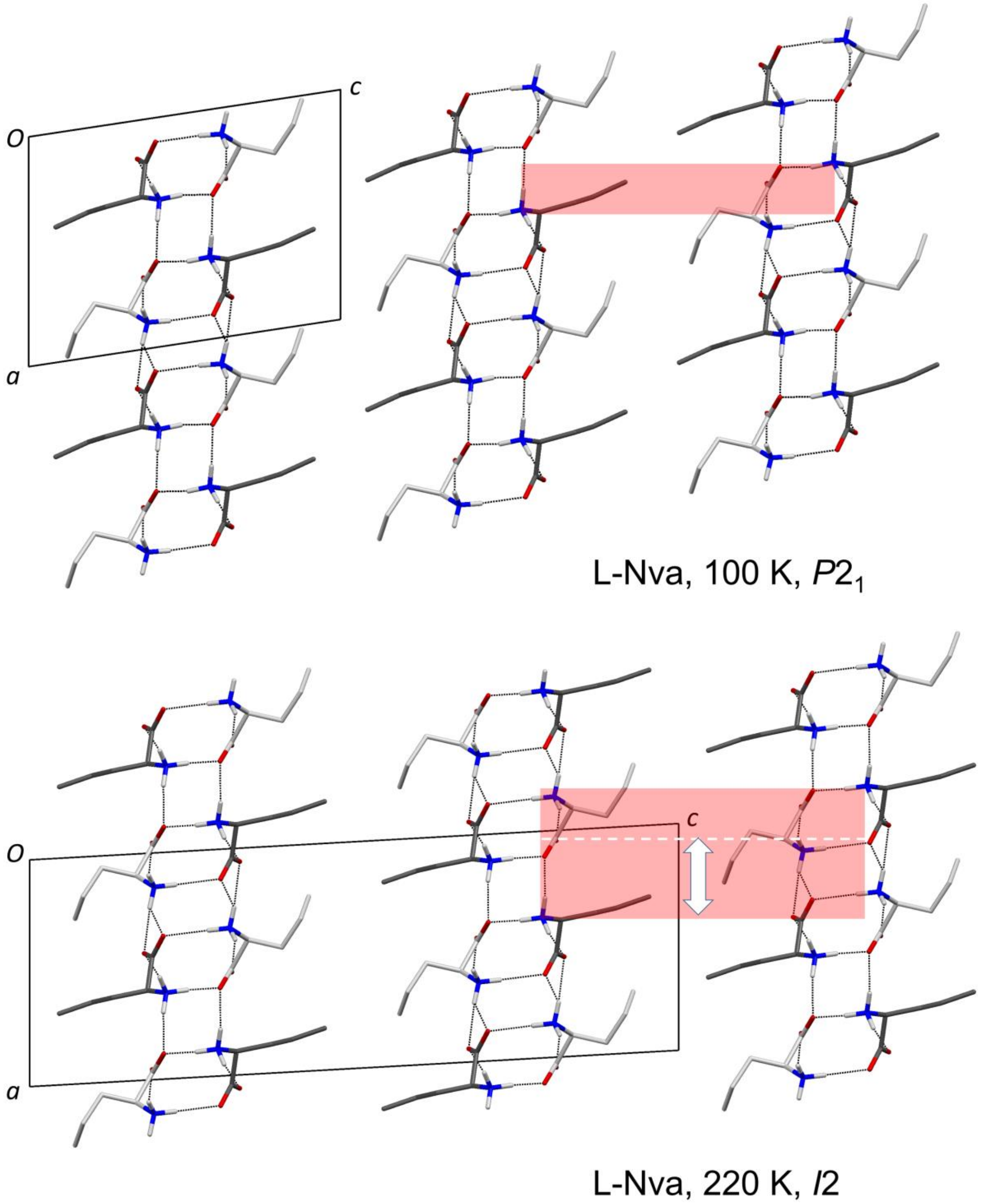

Figure S6 Crystal packing of L-Nva viewed along the *b*-axis at 100 and 220 K. Side-chain hydrogen atoms are omitted, and only the most populated side-chain conformation are shown at 220 K. The red boxes span identical amino groups and illustrate a vertical 3.6 Å shift along the *a*-axis (double arrow) for neighboring molecular bilayers upon transition between the two polymorphs.

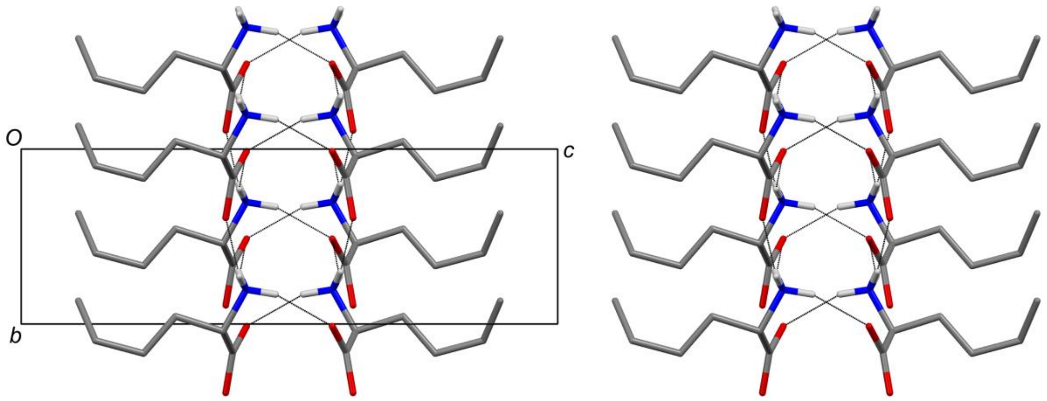

L-Nle, 380 K, *C*2

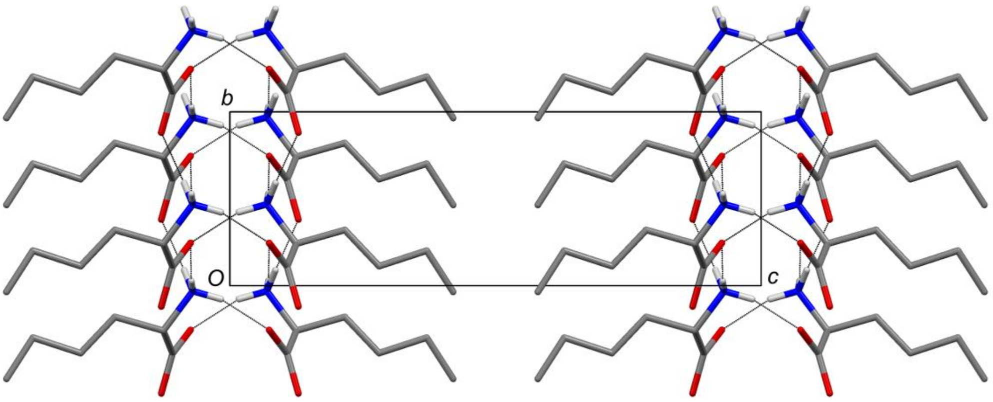

L-Nle, 405 K, *C*2

Figure S7 Crystal packing of L-Nle viewed along the *a*-axis at 380 and 405 K. Side-chain hydrogen atoms are omitted, and only the most populated side-chain conformations are shown. Side-chain conformations are different at the two temperatures, but there is no sliding along the vertical *b*-axis upon transition from one form to the other.

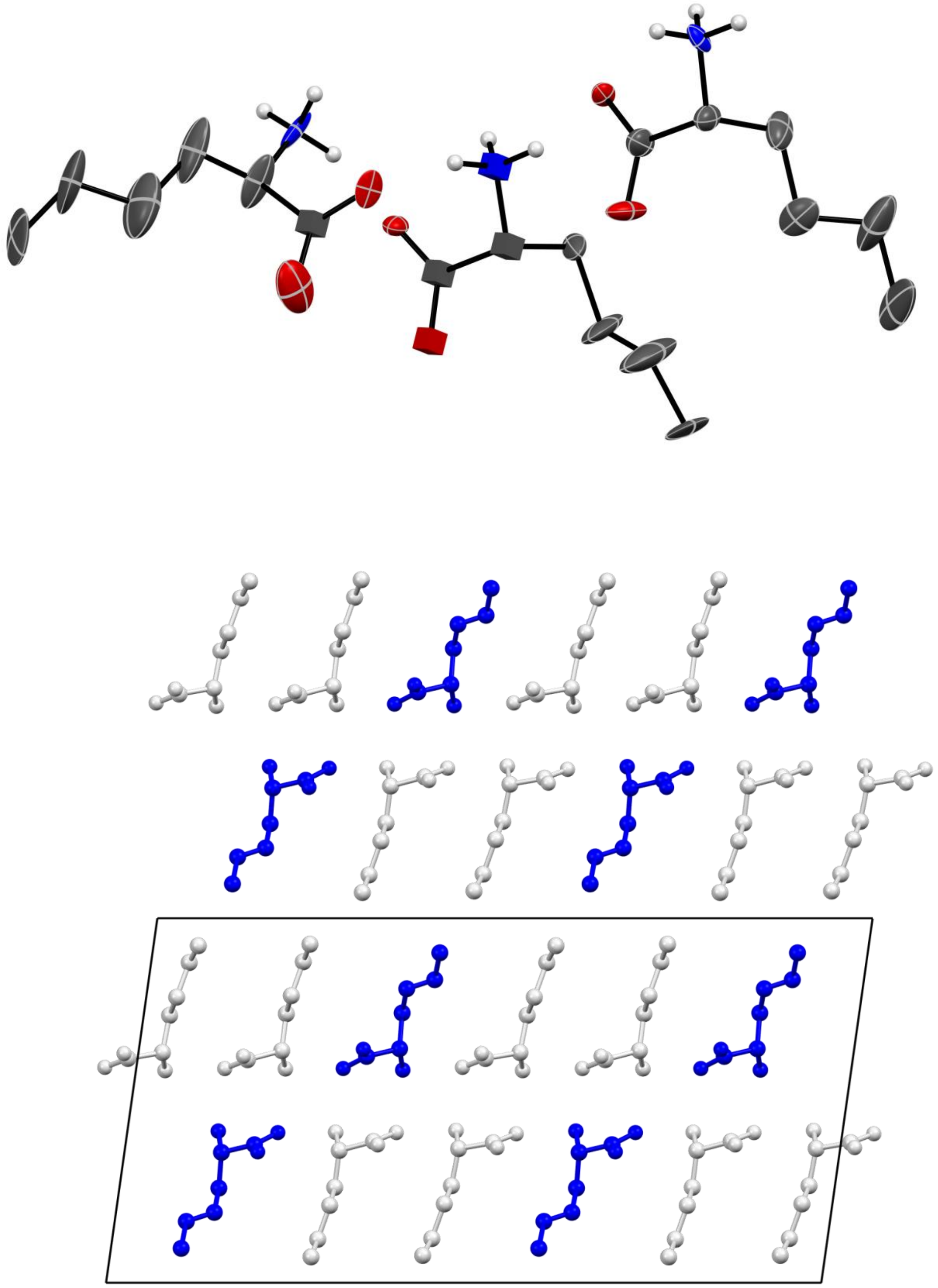

a)

b)

Figure S8 a) Initial asymmetric unit of L-Nle at 180 K, refined in space group *C*2 with *Z*’ = 3. Atoms for which refinement has given non-positive-definite thermal ellipsoids are shown as cubes. b) Unit cell and packing arrangement based on the drawing in a). The molecule with dominating *trans*,*gauche+*,*trans* conformation for the side chain to the right in a), is here colored blue. The type **1** bilayer described in the main body of the paper appears to be dominating. Eventually the structure was shown to be incommensurately modulated.

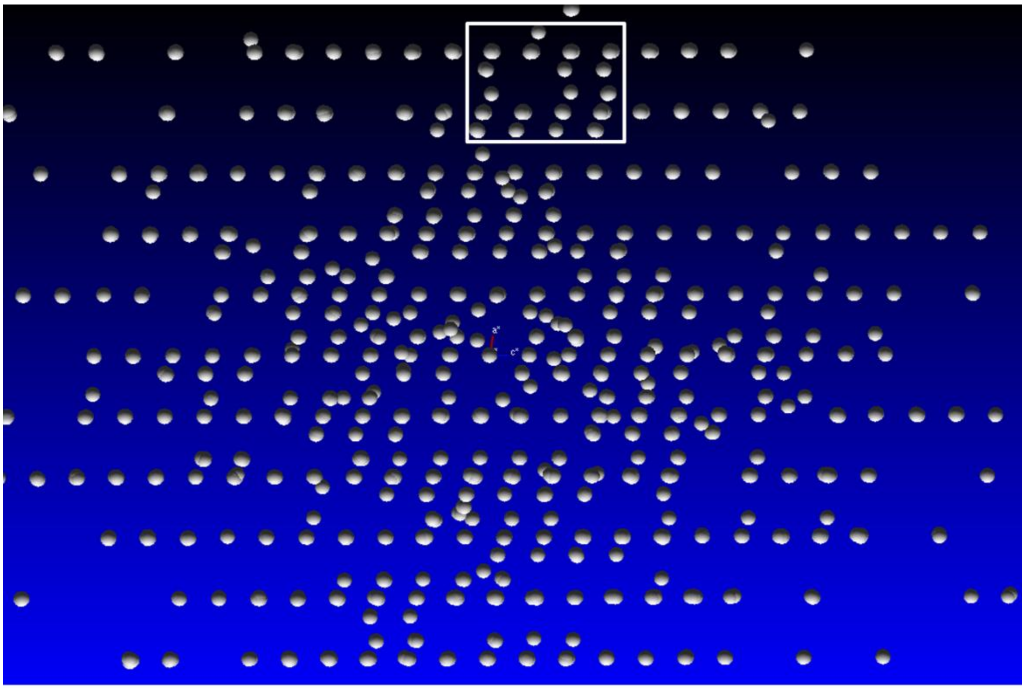

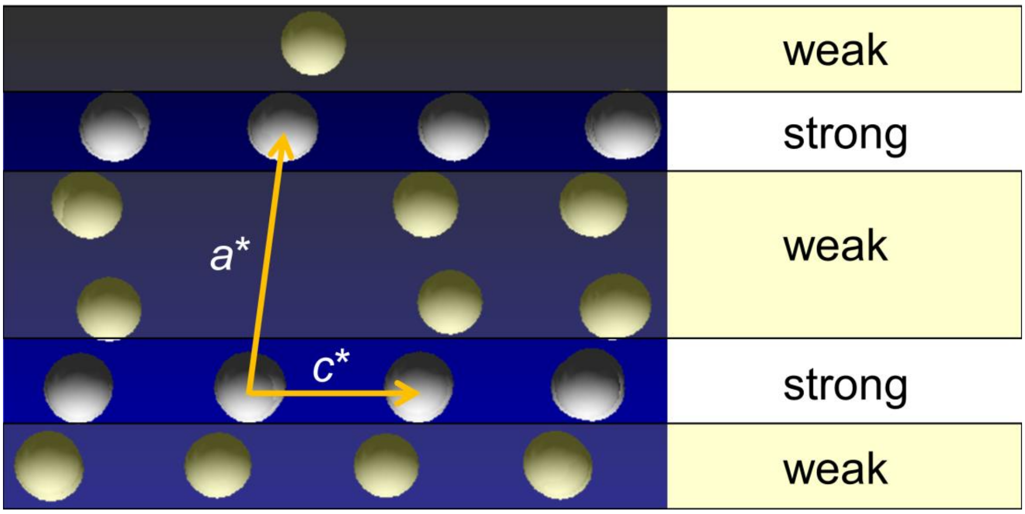

Figure S9 Reciprocal lattice of L-Nle at 180 K viewed along the *b**-axis. The magnified part shows strong reflections defining the *C*2 unit cell at 210 K, with *a** and *c** indicated. Satellite reflections from the modulation are shaded in yellow.

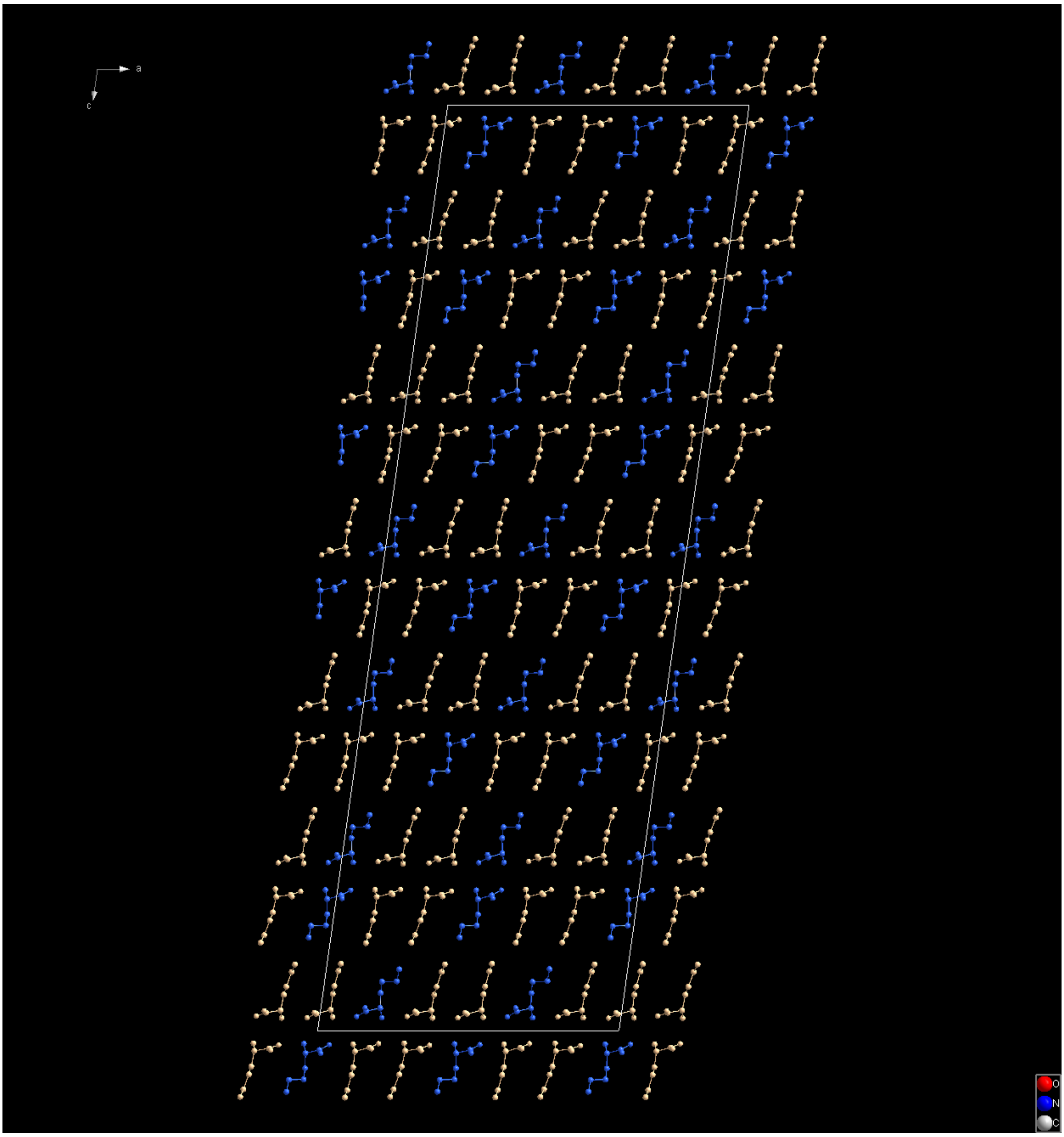
a
c
O
N
C

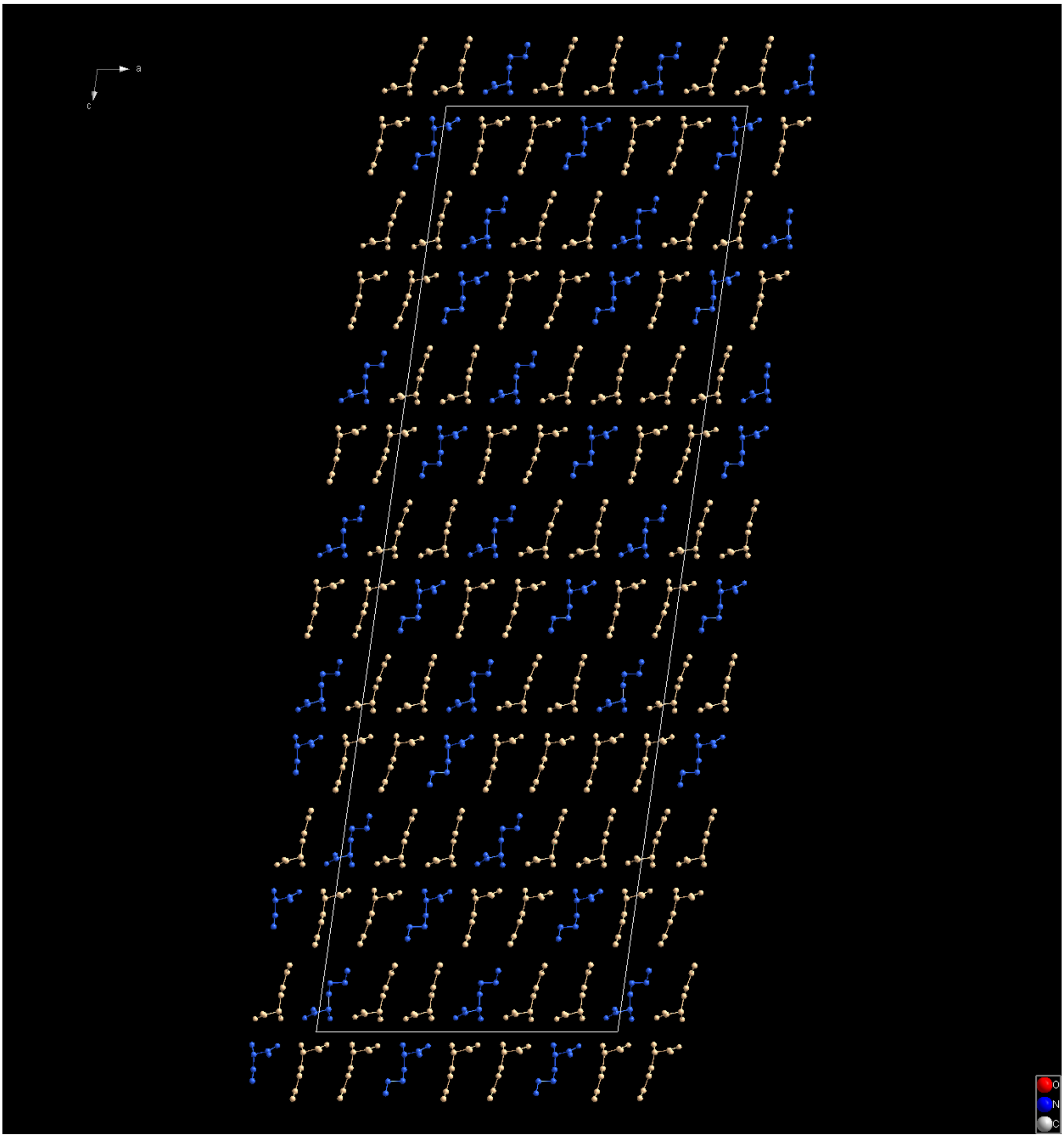

Figure S10 Two figures showing selected sections of the Nle structure at 180 K. Because of the incommensurability there are infinitive numbers of different arrangements. The 3x1x6 supercell from the commensurate 100 K refinement is included also here for comparison, but in this case has no physical significance.

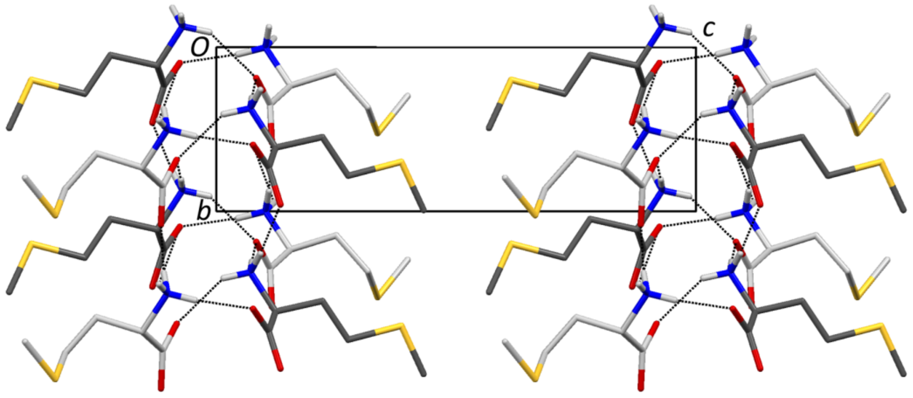

L-Met, 293 K, $P2_1$

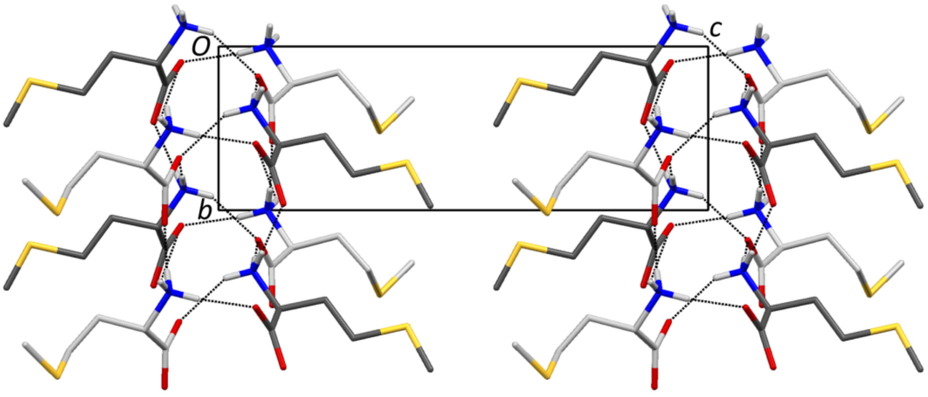

L-Met, 320 K, $P2_1$

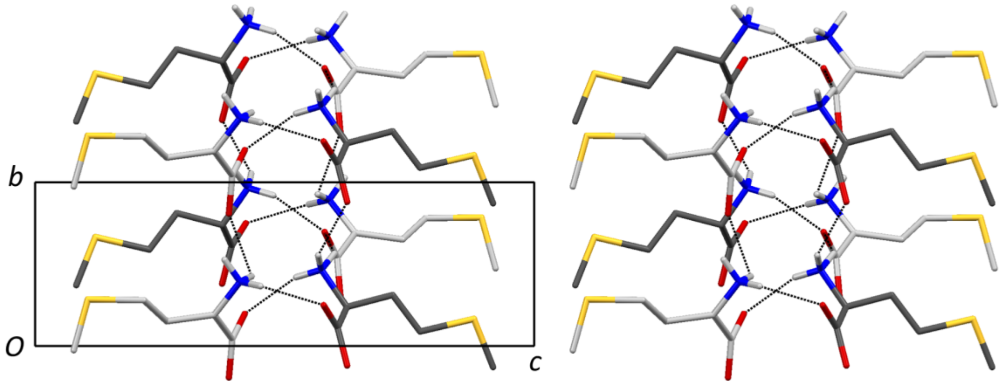

L-Met, 405 K, $P2_1$

Figure S11 Crystal packing of L-Met viewed along the *a*-axis at 293, 320 and 405 K. Side-chain hydrogen atoms are omitted, and only the most populated side-chain conformations are shown. Side-chain conformations are different at the two temperatures, but there is no sliding along the vertical *b*-axis upon transition from one form to the other.

Table S1 Side-chain torsion angles (°) for all refined structures

L-Abu

190 K

N1A C2A C3A C4A -172.8(2)

N1B C21B C31B C41B -57.0(3)

N1B C22B C32B C42B 76(2)

N1C C2C C3C C4C 65.2(3)

N1D C2D C3D C4D -174.11(18)

215 K

N1A C2A C3A C4A -172.1(3)

N11B C21B C31B C41B 63.5(11)

N12B C22B C32B C42B -60.0(14)

N1C C2C C3C C4C 66.9(3)

N1D C2D C3D C4D -174.1(2)

330 K

N11A C21A C31A C41A -169.4(12)

N12A C22A C32A C42A 63(5)

N11B C21B C31B C41B 65.9(18)

N12B C22B C32B C42B -58(3)

N11C C21C C31C C41C 65.3(15)

N12C C22C C32C C42C -171(5)

N1D C2D C3D C4D -173.0(7)

365 K

N11A C21A C31A C41A 66.8(13)

N12A C22A C32A C42A -172(3)

N13A C23A C33A C43A -50(3)

N11B C21B C31B C41B -169.7(14)

N12B C22B C32B C42B 68(2)

N13B C23B C33B C43B -44(5)

L-Nva

100 K

N1A C2A C3A C4A -166.6(3)

C2A C3A C4A C5A 170.1(3)

N1B C2B C3B C4B -64.1(4)

C2B C3B C4B C5B -69.2(4)

190 K

N1A C2A C3A C4A -168.5(3)

C2A C3A C4A C5A 171.0(4)

N1B C2B C3B C4B -65.2(5)

C2B C3B C4B C5B -72.1(6)

220 K

N1A C21A C31A C41A -168.6(8)

C21A C31A C41A C51A 172.1(9)

N1A C22A C32A C42A 86(7)

C22A C32A C42A C52A 174(7)

N1A C23A C33A C43A -175(5)

C23A C33A C43A C53A 76(7)

N11B C21B C31B C41B -64.5(8)

C21B C31B C41B C51B -71.6(11)

N12B C22B C32B C42B -174.0(19)

C22B C32B C42B C52B -179.3(18)

N13B C23B C33B C43B -76(4)

C23B C33B C43B C53B 177(4)

N14B C24B C34B C44B 62(3)

C24B C34B C44B C54B -176.5(19)

270 K

N1A C21A C31A C41A -167.4(5)

C21A C31A C41A C51A 173.7(7)

N1A C22A C32A C42A 71(3)

C22A C32A C42A C52A -171(3)

N1A C23A C33A C43A -166.4(13)

C23A C33A C43A C53A 82(2)

N11B C21B C31B C41B -64.5(9)

C21B C31B C41B C51B -80.5(12)

N12B C22B C32B C42B -178.5(12)

C22B C32B C42B C52B 176.5(15)

N13B C23B C33B C43B -79.3(19)

C23B C33B C43B C53B 162.2(18)

N14B C24B C34B C44B 65.4(14)

C24B C34B C44B C54B -177.0(15)

293 K

N11 C21 C31 C41 -169.7(17)

C21 C31 C41 C51 171.8(18)

N12 C22 C32 C42 65(2)

C22 C32 C42 C52 -178(2)

N13 C23 C33 C43 -55(3)

C23 C33 C43 C53 -176(3)

N14 C24 C34 C44 -172(3)

C24 C34 C44 C54 73(3)

L-Nle

210 K

N11 C21 C31 C41 -168.1(6)

C21 C31 C41 C51 170.5(5)

C31 C41 C51 C61 -177.2(7)

N12 C22 C32 C42 -164.7(13)

C22 C32 C42 C52 71.5(14)

C32 C42 C52 C62 170.7(13)

330 K

N11 C21 C31 C41 -169.1(8)

C21 C31 C41 C51 175.9(11)

C31 C41 C51 C61 -173.4(14)

N12 C22 C32 C42 -169(3)

C22 C32 C42 C52 76(3)

C32 C42 C52 C62 94(5)

380 K

N11 C21 C31 C41 -170(2)

C21 C31 C41 C51 174(2)

C31 C41 C51 C61 -174(3)

N12 C22 C32 C42 66(4)

C22 C32 C42 C52 -172(3)

C32 C42 C52 C62 -79(4)

N13 C23 C33 C43 -46(4)

C23 C33 C43 C53 -179.4(9)

C33 C43 C53 C63 80(4)

405 K

N1 C21 C31 C41 -179.0(16)

C21 C31 C41 C51 179(2)

C31 C41 C51 C61 46(6)

N1 C22 C32 C42 -66(7)

C22 C32 C42 C52 -179(2)

C32 C42 C52 C62 64(6)

N1 C23 C33 C43 61(6)

C23 C33 C43 C53 -179.2(18)

C33 C43 C53 C63 60(6)

N1 C24 C34 C44 178.4(18)

C24 C34 C44 C54 178(3)

C34 C44 C54 C64 -178(3)

L-Met

293 K

N1A C21A C31A C41A -165.6(3)

C21A C31A C41A S11A 172.7(3)

C31A C41A S11A C51A -177.6(5)

N1A C22A C32A C42A -162.9(11)

C22A C32A C42A S12A 178.9(9)

C32A C42A S12A C52A 179.7(8)

N1B C21B C31B C41B -166.4(4)

C21B C31B C41B S11B 72.6(6)

C31B C41B S11B C51B 72.7(5)

N1B C22B C32B C42B -70(2)

C22B C32B C42B S12B -74(2)

C32B C42B S12B C52B -70.1(13)

N1B C23B C33B C43B -155(2)

C23B C33B C43B S13B -179(2)

C33B C43B S13B C53B -63(2)

N1B C24B C34B C44B -56(4)

C24B C34B C44B S14B -165(3)

C34B C44B S14B C54B 76(3)

320 K

N1A C21A C31A C41A 174.9(11)

C21A C31A C41A S11A -179.6(9)

C31A C41A S11A C51A 179.3(9)

N1A C22A C32A C42A -155.2(6)

C22A C32A C42A S12A 167.1(5)

C32A C42A S12A C52A 165.0(9)

N1B C21B C31B C41B -164.1(8)

C21B C31B C41B S11B 73.4(11)

C31B C41B S11B C51B 76.8(8)

N1B C22B C32B C42B -72.9(13)

C22B C32B C42B S12B -71.3(13)

C32B C42B S12B C52B -78.9(10)

N1B C23B C33B C43B -132.0(16)

C23B C33B C43B S13B 173.5(16)

C33B C43B S13B C53B -62.7(15)

N1B C24B C34B C44B -88.7(18)

C24B C34B C44B S14B -159.2(16)

C34B C44B S14B C54B 66.4(18)

405 K

N1A C21A C31A C41A -174.0(18)

C21A C31A C41A S11A 176.8(16)

C31A C41A S11A C51A 87(5)

N1A C22A C32A C42A -168.1(13)

C22A C32A C42A S12A 173.8(13)

C32A C42A S12A C52A -178.4(14)

N1A C23A C33A C43A -47(8)

C23A C33A C43A S13A -178.5(17)

C33A C43A S13A C53A -70(6)

N1A C24A C34A C44A 69(6)

C24A C34A C44A S14A -179.3(17)

C34A C44A S14A C54A 71(6)

N1B C21B C31B C41B -179.0(15)

C21B C31B C41B S11B 66(5)

C31B C41B S11B C51B 65(6)

N1B C22B C32B C42B -82(4)

C22B C32B C42B S12B -78(4)

C32B C42B S12B C52B -61(5)

N1B C23B C33B C43B 48(9)

C23B C33B C43B S13B -179.4(12)

C33B C43B S13B C53B -61(10)

N1B C24B C34B C44B -40(7)

C24B C34B C44B S14B 172(4)

C34B C44B S14B C54B 59(5)

N1B C25B C35B C45B -179.1(13)

C25B C35B C45B S15B 179.8(12)

C35B C45B S15B C55B -57(6)

N1B C26B C36B C46B -178.2(13)

C26B C36B C46B S16B 178.9(12)

C36B C46B S16B C56B -64(5)

Table S2 Hydrogen-bond geometries (Å,°) in structures where amino acid polar heads were refined without disorder or with a component with occupancy > 0.69

Interaction	N-H	H···O	N···O	N-H···O	Hydrogen bond[a]
L-Abu 110 K, *I*2[b]					
N1A–H1A···O1A	0.91	1.89	2.775(4)	162.5	3
N1A–H2A···O1B	0.91	1.94	2.817(4)	160.3	1
N1A–H2A···O2B	0.91	2.50	3.255(4)	141.0	1
N1A–H3A···O2A	0.91	1.87	2.768(4)	168.1	2
N1B–H1B···O1B	0.91	1.91	2.798(4)	163.2	3
N1B–H2B···O1A	0.91	1.92	2.800(4)	162.5	1
N1B–H2B···O2A	0.91	2.59	3.323(4)	138.3	1
N1B–H3B···O2B	0.91	1.87	2.773(4)	172.8	2
N1C–H1C···O1D	0.91	1.98	2.822(4)	154.0	3
N1C–H2C···O2C	0.91	1.92	2.804(4)	163.9	2
N1C–H3C···O1D	0.91	1.94	2.823(4)	162.9	1
N1C–H3C···O2D	0.91	2.50	3.230(4)	137.3	1
N1D–H1D···O1C	0.91	1.95	2.818(4)	159.4	1
N1D–H1D···O2C	0.91	2.50	3.260(4)	141.5	1
N1D–H2D···O2D	0.91	1.87	2.755(4)	164.3	2
N1D–H3D···O1C	0.91	1.91	2.779(4)	159.7	3
L-Abu 190 K, *I*2					
N1A–H1A···O1A	0.91	1.89	2.770(2)	162.3	3
N1A–H2A···O1B	0.91	1.95	2.819(2)	159.3	1
N1A–H2A···O2B	0.91	2.47	3.233(2)	141.8	1
N1A–H3A···O2A	0.91	1.87	2.767(2)	168.1	2
N1B–H1B···O1B	0.91	1.91	2.794(2)	162.3	3
N1B–H2B···O1A	0.91	1.91	2.792(2)	161.9	1
N1B–H2B···O2A	0.91	2.56	3.305(2)	139.1	1
N1B–H3B···O2B	0.91	1.87	2.775(2)	173.9	2
N1C–H1C···O2C	0.91	1.93	2.812(2)	164.2	2

N1C–H2C···O1D	0.91	1.99	2.829(2)	153.2	3
N1C–H3C···O1D	0.91	1.91	2.802(2)	164.9	1
N1C–H3C···O2D	0.91	2.53	3.240(2)	135.4	1
N1D–H1D···O2D	0.91	1.87	2.756(2)	162.6	2
N1D–H2D···O1C	0.91	1.90	2.772(2)	161.0	3
N1D–H3D···O1C	0.91	1.97	2.830(2)	156.5	1
N1D–H3D···O2C	0.91	2.44	3.217(2)	143.3	1
L-Abu 215 K, $P2_1$					
N1A–H1A···O11B	0.90	1.90	2.78(3)	165.9	3
N1A–H2A···O11B	0.90	2.05	2.884(16)	154.1	1
N1A–H2A···O21B	0.90	2.38	3.17(2)	146.3	1
N1A–H3A···O2A	0.90	1.88	2.752(3)	162.7	2
N11B–H1B···O1A	0.90	1.98	2.778(14)	146.3	3
N11B–H2B···O1A	0.90	1.89	2.770(15)	164.1	1
N11B–H2B···O2A	0.90	2.53	3.25(2)	137.7	1
N11B–H3B···O21B	0.90	2.02	2.90(6)	164.4	2
N1C–H1C···O1D	0.90	1.88	2.773(3)	168.5	1
N1C–H1C···O2D	0.90	2.61	3.284(3)	132.4	1
N1C–H2C···O2C	0.90	1.95	2.827(3)	165.2	2
N1C–H3C···O1D	0.90	2.05	2.867(3)	151.1	3
N1D–H1D···O1C	0.90	2.06	2.873(3)	150.4	1
N1D–H1D···O2C	0.90	2.35	3.156(3)	148.4	1
N1D–H2D···O2D	0.90	1.91	2.771(3)	159.7	2
N1D–H3D···O1C	0.90	1.89	2.760(3)	163.5	3
L-Nva 100 K, $P2_1$					
N1A–H1A···O1B	0.91	1.84	2.744(4)	173.3	1
N1A–H2A···O2A	0.91	1.94	2.839(4)	171.3	2
N1A–H3A···O1B	0.91	2.07	2.824(4)	139.5	3
N1B–H1B···O1A	0.91	1.90	2.799(4)	171.3	3
N1B–H2B···O2A	0.91	1.93	2.829(3)	169.6	1

N1B–H2B···O1A	0.91	2.47	3.085(3)	125.2	1
N1B–H3B···O2B	0.91	1.94	2.823(4)	163.8	2
L-Nva 190 K, $P2_1$					
N1A–H1A···O1B	0.91	1.84	2.745(4)	176.6	1
N1A–H2A···O2A	0.91	1.94	2.846(4)	174.7	2
N1A–H3A···O1B	0.91	2.07	2.818(4)	138.9	3
N1B–H1B···O1A	0.91	1.89	2.798(4)	171.7	3
N1B–H2B···O2A	0.91	1.95	2.850(4)	168.3	1
N1B–H2B···O1A	0.91	2.47	3.084(4)	125.3	1
N1B–H3B···O2B	0.91	1.94	2.825(4)	164.6	2
L-Nva 220 K, *I*2					
N1A–H1A···O11B	0.90	1.87	2.761(15)	169.9	1
N1A–H2A···O2A	0.90	1.97	2.852(3)	166.3	2
N1A–H3A···O11B	0.90	2.00	2.797(18)	146.7	3
N11B–H1B···O2A[c]	0.90	1.96	2.855(19)	171.5	1
N11B–H1B···O1A	0.90	2.41	3.019(12)	124.7	1
N11B–H2B···O21B	0.90	1.99	2.84(3)	158.4	2
N11B–H3B···O1A	0.90	1.96	2.858(13)	177.6	3
L-Nva 293 K, C2[d]					
N11–H1···O11	0.89	1.95	2.828(19)	166.9	1
N11–H1···O21	0.89	2.62	3.32(5)	136.9	1
N11–H2···O21	0.89	2.04	2.92(5)	170.8	2
N11–H3···O11	0.89	1.71	2.56(4)	157.0	3
L-Nle 210 K, *C*2					
N11–H1···O11	0.90	1.96	2.80(2)	155.5	3
N11–H2···O11	0.90	1.91	2.793(9)	166.8	1
N11–H2···O21	0.90	2.60	3.296(16)	135.1	1
N11–H3···O21	0.90	1.90	2.769(16)	162.5	2
L-Nle 330 K, *C*2					
N11–H1···O11	0.89	1.96	2.790(16)	155.1	3

N11–H2···O11	0.89	1.98	2.837(6)	161.6	1
N11–H2···O21	0.89	2.50	3.231(15)	140.4	1
N11–H3···O21	0.89	1.92	2.786(13)	164.6	2
L-Met 293 K, $P2_1$					
N1A–H1A···O1B	0.89	2.08	2.856(3)	145.3	3
N1A–H2A···O1B	0.89	1.84	2.732(3)	176.9	1
N1A–H3A···O2A	0.89	1.98	2.856(3)	169.4	2
N1B–H1B···O1A	0.89	1.90	2.769(3)	166.3	3
N1B–H2B···O2A	0.89	2.05	2.881(3)	155.2	1
N1B–H2B···O1A	0.89	2.32	2.991(3)	131.6	1
N1B–H3B···O2B	0.89	1.97	2.854(4)	173.1	2
L-Met 320 K, $P2_1$					
N1A–H1A···O1B	0.89	2.05	2.853(5)	149.6	3
N1A–H2A···O1B	0.89	1.85	2.738(4)	173.1	1
N1A–H3A···O2A	0.89	1.98	2.857(5)	166.2	2
N1B–H1B···O1A	0.89	1.92	2.785(5)	163.7	3
N1B–H2B···O2A	0.89	2.09	2.908(4)	152.7	1
N1B–H2B···O1A	0.89	2.32	3.000(4)	132.7	1
N1B–H3B···O2B	0.89	1.96	2.850(5)	172.7	2

[a] Hydrogen–bond numbers 1 and 2 appear in Figure 6, hydrogen bond 3 connects two sheets into a layer, as seen in e.g. Figure 3.
[b] (Görbitz, 2010)
[c] Occupancy for orientation 1 is 0.528(3).
[d] Occupancy for orientation 1 is 0.369(14).

Table S3 Experimental and refinement details for L-Nle at 180K

	Harmonic model	Crenel model
Chemical formula	$C_6H_{13}NO_2$	
Temperature	180K	
Cell setting	Monoclinic	
Superspace group	$C2(\alpha,0,\gamma)0$	
a/Å	9.5223(3)	
b/Å)	5.24520(10)	
c/Å	14.7504(4)	
β/°	98.012(2)	
V/Å^3	729.54(3)	
Formula units	4	
D_x /Mg m^{-3}	1.1943	
Modulation vector	0.6978(10)$\mathbf{a}^*$ - 0.1095$\mathbf{c}^*$(13)	
Crystal form	plate	
Crystal size /mm	0.68 x 0.58 x 0.16	
Crystal color	colorless	
Diffractometer	D8 Vantage single crystal CCD-diffractometer	
Radiation type	Mo K_α	
Wavelength /Å	0.71069	
Absorption correction type	Spherical harmonics	
Absorption coefficient μ /mm^{-1}	0.089	
T_{min}/T_{max}	0.597/1	
Range of *h,k,l,m*	$-13 \rightarrow h \rightarrow 13$<br>$-7 \rightarrow k \rightarrow 7$<br>$-20 \rightarrow l \rightarrow 20$<br>$-3 \rightarrow m \rightarrow 3$	
No. of measured reflections	29709	
No. of unique reflections	11209	
No. of observed reflections	2905	
No. of main reflections	1612	
No. of satellite reflections	9597	
Criterion for observed reflections	$I > 2\sigma(I)$	
R_{int} (obs) all reflections	0.0405	
R_{int} (obs) main reflections	0.0289	
R_{int} (obs) 1st order satellites	0.1174	
R_{int} (obs) 2nd order satellites	0.2918	
R_{int} (obs) 3rd order satellites	0.4781	
Refinement on	F^2	
R, *wR* (all reflections)	0.0851/0.1272	0.0992/0.1547
R, *wR* (main reflections)	0.0511/0.0851	0.0635/0.1082
R, *wR* (1st order satellites)	0.1240/0.2162	0.1415/0.2503
R, *wR* (2nd order satellites)	0.2696/0.3605	0.3002/0.5110
R, *wR* (3rd order satellites)	0.8796/0.9735	0.8234/0.9348
GOF	1.02	1.22
No. of parameters	170	167
$\Delta\rho_{max}$ (eÅ^{-3})	1.01	1.24
$\Delta\rho_{min}$ (eÅ^{-3})	-1.09	-0.98
Extinction correction	none	
Source of atomic scattering factors	*International Tables for X-ray for Crystallography* (1992 Vol.C)	
Program – refinement	Jana2006	

Table S4 Experimental and refinement details for L-Nle at 100 K

	Harmonic model	Crenel model
Chemical formula	$C_6H_{13}NO_2$	
Temperature	100K	
Cell setting	Monoclinic	
Superspace group	$C2(\alpha,0,\gamma)0$	
a/Å	9.5245(10)	
b/Å	5.2348(6)	
c/Å	14.6489(16)	
β/°	98.741(9)	
V/Å^3	721.89(14)	
Formula units	4	
D_x /Mg m^{-3}	1.2069	
Modulation vector	2/3$\mathbf{a}^*$ - 1/6$\mathbf{c}^*$	
Crystal form	plate	
Crystal size /mm	0.68 x 0.58 x 0.16	
Crystal color	colorless	
Diffractometer	D8 Vantage single crystal CCD-diffractometer	
Radiation type	Mo K_α	
Wavelength /Å	0.71069	
Absorption correction type	Spherical harmonics	
Absorption coefficient μ /mm^{-1}	0.09	
T_{min}/T_{max}	0.327/1	
Range of *h*,*k*,*l*,*m*	$-11 \rightarrow h \rightarrow 13$	
	$-6 \rightarrow k \rightarrow 6$	
	$-20 \rightarrow l \rightarrow 20$	
	$-3 \rightarrow m \rightarrow 3$	
No. of measured reflections	34691	
No. of unique reflections	9704	
No. of observed reflections	3946	
No. of main reflections	1627	
No. of satellite reflections	8077	
Criterion for observed reflections	$I > 2\sigma(I)$	
R_{int}(obs) all reflections	0.0957	
R_{int}(obs) main reflections	0.0756	
R_{int}(obs) 1st order satellites	0.1455	
R_{int}(obs) 2nd order satellites	0.2533	
R_{int}(obs) 3rd order satellites	0.5007	
Refinement on	F^2	
R, *wR* (all reflections)	0.1162/0.1802	0.1187/0.1875
R, *wR* (main reflections)	0.0706/0.1223	0.0733/0.1270
R, *wR* (1st order satellites)	0.1278/0.2305	0.1333/0.2419
R, *wR* (2nd order satellites)	0.2035/0.3371	0.1988/0.3565
R, *wR* (3rd order satellites)	0.6826/0.8666	0.6649/0.8566
GOF	1.38	1.43
No. of parameters	191	186
$\Delta\rho_{max}$ (eÅ^{-3})	1.28	1.34
$\Delta\rho_{min}$ (eÅ^{-3})	-0.97	-0.94
Extinction correction	none	

Source of atomic scattering factors	*International Tables for X-ray for Crystallography* (1992 Vol.C)
Program – refinement	Jana2006

Table S5 Molecular geometry (Å, °) of the two L-Nle conformations at 210, 180 and 100 K.

trans,trans,trans

distance/angle/ torsion angle	210K	180K		100K	
		Crenel	Harmonic	Crenel	Harmonic
O1-C1	1.259(3)	1.255(13)	1.261(15)	1.269(6)	1.273(7)
O2-C1	1.239(3)	1.245(15)	1.243(17)	1.244(7)	1.242(9)
N1-C2	1.485(4)	1.486(18)	1.48(2)	1.492(8)	1.492(11)
C1-C2	1.531(4)	1.528(13)	1.529(16)	1.525(7)	1.526(8)
C2-C3	1.527(4)	1.515(14)	1.526(16)	1.526(7)	1.529(9)
C3-C4	1.528(6)	1.54(2)	1.53(2)	1.533(9)	1.525(12)
C4-C5	1.518(5)	1.461(16)	1.506(19)	1.526(8)	1.539(10)
C5-C6	1.529(8)	1.53(3)	1.53(2)	1.532(11)	1.534(15)
O1-C1-O2	124.3(3)	124.5(9)	124.8(11)	124.1(5)	124.2(6)
O1-C1-C2	116.9(3)	117.0(9)	116.4(11)	116.7(5)	116.5(6)
O2-C1-C2	118.8(4)	118.4(9)	118.7(11)	119.1(5)	119.3(6)
N1-C2-C1	109.3(5)	108.8(8)	109.3(9)	109.1(4)	109.1(5)
N1-C2-C3	109.6(3)	110.3(10)	110.0(12)	109.5(5)	109.3(7)
C1-C2-C3	111.9 (3)	113.1(7)	112.6(8)	113.1(4)	113.0(5)
C2-C3-C4	113.1(3)	112.9(11)	113.1(13)	112.3(5)	112.5(7)
C3-C4-C5	112.7(4)	113.4(12)	112.4(14)	112.3(6)	112.2(8)
C4-C5-C6	112.1(6)	112.8(14)	111.6(16)	111.8(6)	111.8(9)
O1-C1-C2-N1	-19.7(15)	-24.0(6)	-23.4(8)	-22.4(4)	-22.7(5)
O2-C1-C2-N1	161.8(15)	159.0(6)	158.9(7)	160.5(4)	160.4(5)
O1-C1-C2-C3	101.7(14)	99.0(6)	99.1(7)	99.7(4)	99.2(5)
O2-C1-C2-C3	-76.8(15)	-78.1(6)	-78.6(8)	-77.3(5)	-77.7(5)
N1-C2-C3-C4	-167.2(8)	-166.3(5)	-166.8(7)	-166.2(3)	-166.7(4)
C2-C3-C4-C5	170.5(6)	169.9(6)	170.8(8)	172.2(4)	172.6(4)
C3-C4-C5-C6	-176.9(7)	-176.1(7)	-176.6(9)	-176.7(4)	-176.8(5)

trans,gauche+,trans

distance/angle/ torsion angle	210K	180K		100K	
		Crenel	Harmonic	Crenel	Harmonic
O1-C1	1.258(5)	1.26(2)	1.26(5)	1.269(7)	1.269(8)
O2-C1	1.239(5)	1.24(3)	1.24(7)	1.245(9)	1.246(10)
N1-C2	1.486(5)	1.48(3)	1.47(8)	1.472(8)	1.472(10)
C1-C2	1.532(5)	1.55(2)	1.55(5)	1.520(8)	1.521(9)
C2-C3	1.525(6)	1.51(3)	1.52(6)	1.533(7)	1.533(8)
C3-C4	1.527(7)	1.53(3)	1.51(8)	1.531(9)	1.525(10)
C4-C5	1.518(7)	1.46(4)	1.55(8)	1.502(8)	1.514(10)
C5-C6	1.527(9)	1.56(4)	1.52(10)	1.550(9)	1.541(11)
O1-C1-O2	124.5(6)	125.0(18)	125(4)	124.2(6)	124.3(6)
O1-C1-C2	116.8(5)	116.2(18)	116(4)	116.7(6)	117.0(6)
O2-C1-C2	118.7(5)	118.7(18)	118(4)	119.1(6)	118.7(6)
N1-C2-C1	109.2(5)	109.6(15)	110(3)	110.6(5)	110.4(5)
N1-C2-C3	109.4(6)	108.4(19)	109(5)	107.4(5)	107.6(6)
C1-C2-C3	112.0(6)	111.4(13)	111(3)	111.0(4)	111.3(5)
C2-C3-C4	113.7(6)	117(2)	116(5)	115.9(6)	115.6(7)
C3-C4-C5	112.7(6)	113.0(18)	113(4)	112.9(5)	113.0(6)
C4-C5-C6	112.6(8)	114(2)	115(4)	113.9(5)	114.2(6)
O1-C1-C2-N1	-33(4)	-25.7(11)	-27(3)	-24.4(6)	-24.5(6)

O2-C1-C2-N1	147(4)	156.6(10)	157(3)	158.1(5)	158.2(5)
O1-C1-C2-C3	88(4)	94.2(10)	94(3)	94.7(6)	94.9(6)
O2-C1-C2-C3	-91(4)	-83.5(11)	-83(3)	-82.8(6)	-82.4(7)
N1-C2-C3-C4	-167.4(18)	-170.5(10)	-168(2)	-166.8(4)	-166.6(4)
C2-C3-C4-C5	71.2(16)	77.1(12)	71(3)	72.0(6)	71.6(6)
C3-C4-C5-C6	171.5(14)	175.0(14)	174(4)	175.2(5)	174.8(6)

Table S6 Molecular geometry (Å, °) of the two L-Nle conformations at 100 K for three refinement models

trans,trans,trans

Distance/angle/ torsion angle	1st rigid body refinement	Atomic refinement				2nd rigid body refinement	
	B+C+E+F	B	C	E	F	B+E	C+F
O1-C1	1.269(6)	1.263(8)	1.264(8)	1.278(8)	1.274(8)	1.267(7)	1.273(7)
O2-C1	1.244(7)	1.253(7)	1.251(7)	1.235(7)	1.238(7)	1.243(7)	1.241(7)
N1-C2	1.492(8)	1.488(8)	1.481(7)	1.486(7)	1.486(7)	1.489(7)	1.484(7)
C1-C2	1.525(7)	1.538(9)	1.520(9)	1.522(9)	1.525(9)	1.532(8)	1.519(8)
C2-C3	1.526(7)	1.551(7)	1.553(7)	1.512(8)	1.514(7)	1.537(7)	1.533(7)
C3-C4	1.533(9)	1.527(8)	1.549(8)	1.543(8)	1.515(9)	1.532(8)	1.536(8)
C4-C5	1.526(8)	1.523(8)	1.506(8)	1.534(8)	1.541(8)	1.528(8)	1.521(8)
C5-C6	1.532(11)	1.520(9)	1.536(9)	1.541(9)	1.529(9)	1.530(9)	1.536(9)
O1-C1-O2	124.1(5)	124.6(6)	124.1(6)	124.2(6)	124.5(6)	124.6(6)	124.3(6)
O1-C1-C2	116.7(5)	117.3(5)	116.8(5)	116.3(5)	115.8(5)	116.9(5)	116.0(5)
O2-C1-C2	119.1(5)	118.1(6)	119.0(6)	119.4(6)	119.6(6)	118.5(5)	119.6(6)
N1-C2-C1	109.1(4)	109.0(5)	109.2(5)	110.6(5)	109.3(5)	109.8(5)	109.5(5)
N1-C2-C3	109.5(5)	109.6(5)	109.8(4)	108.6(5)	109.0(5)	108.9(5)	109.3(5)
C1-C2-C3	113.1(4)	112.5(4)	112.7(4)	113.2(4)	112.0(4)	112.8(4)	112.4(4)
C2-C3-C4	112.3(5)	112.3(5)	111.9(5)	112.1(5)	113.4(5)	112.2(5)	112.6(5)
C3-C4-C5	112.3(6)	111.7(4)	111.4(5)	113.0(5)	113.4(5)	112.3(5)	112.2(5)
C4-C5-C6	111.8(6)	112.4(5)	111.8(5)	110.9(5)	112.7(5)	111.6(5)	111.9(5)
O1-C1-C2-N1	-22.4(4)	-19.3(5)	-25.2(5)	-17.9(5)	-25.1(5)	-18.3(4)	-25.1(4)
O2-C1-C2-N1	160.5(4)	162.2(4)	158.2(4)	164.3(4)	157.9(4)	163.5(3)	158.1(3)
O1-C1-C2-C3	99.7(4)	102.5(5)	97.1(5)	104.2(5)	95.8(5)	103.3(4)	96.6(4)
O2-C1-C2-C3	-77.3(5)	-76.0(6)	-79.5(6)	-73.7(6)	-81.2(6)	-74.9(5)	-80.2(5)
N1-C2-C3-C4	-166.2(3)	-165.4(5)	-167.2(5)	-162.8(4)	-168.2(5)	121.7(5)	121.7(5)
C2-C3-C4-C5	172.2(4)	171.8(4)	171.2(5)	172.9(5)	172.2(5)	172.3(4)	171.7(4)
C3-C4-C5-C6	-176.7(4)	-176.5(5)	-176.7(5)	-177.3(5)	-177.3(5)	-176.9(4)	-177.3(4)

trans,gauche+,trans

Distance/angle/ torsion angle	1st rigid body refinement	Atomic refinement		2nd rigid body refinement
	A+D	A	D	A+D
O1-C1	1.269(8)	1.269(8)	1.268(8)	1.266(7)
O2-C1	1.246(10)	1.247(7)	1.240(7)	1.243(7)

N1-C2	1.472(10)	1.490(7)	1.483(7)	1.485(7)
C1-C2	1.521(9)	1.514(9)	1.520(9)	1.517(9)
C2-C3	1.533(8)	1.548(7)	1.526(7)	1.535(7)
C3-C4	1.525(10)	1.527(8)	1.533(8)	1.528(8)
C4-C5	1.514(10)	1.494(9)	1.506(9)	1.502(9)
C5-C6	1.541(11)	1.529(8)	1.550(8)	1.533(9)
O1-C1-O2	124.3(6)	123.9(6)	124.2(6)	124.0(6)
O1-C1-C2	117.0(6)	117.4(5)	116.6(5)	116.9(5)
O2-C1-C2	118.7(6)	118.7(6)	119.1(6)	119.0(6)
N1-C2-C1	110.4(5)	109.9(5)	109.6(5)	109.9(5)
N1-C2-C3	107.6(6)	107.8(5)	106.5(5)	107.3(4)
C1-C2-C3	111.3(5)	111.5(4)	111.1(4)	111.2(4)
C2-C3-C4	115.6(7)	114.9(5)	115.2(5)	115.2(5)
C3-C4-C5	113.0(6)	113.9(4)	113.4(4)	114.1(4)
C4-C5-C6	114.2(6)	114.1(4)	113.4(4)	113.8(4)
O1-C1-C2-N1	-24.5(6)	-24.9(6)	-24.7(5)	-24.8(4)
O2-C1-C2-N1	158.2(5)	158.4(4)	157.5(4)	157.8(3)
O1-C1-C2-C3	94.9(6)	94.6(6)	92.8(5)	93.9(4)
O2-C1-C2-C3	-82.4(7)	-82.1(6)	-85.1(6)	-83.5(5)
N1-C2-C3-C4	-166.6(4)	-166.8(5)	-165.6(5)	118.6(5)
C2-C3-C4-C5	71.6(6)	72.0(7)	69.8(7)	70.6(6)
C3-C4-C5-C6	174.8(6)	174.9(5)	175.3(5)	174.9(5)

	1st rigid body refinement	Atomic refinement	2nd rigid body refinement
R, *wR* (observed)	0.1187/0.1884	0.0963/0.1426	0.1001/0.1520
GOF	1.43	1.19	1.24
No. of parameters	186	271	208

1st rigid body refinement: Similar geometries for A+D and for B+C+E+F

2nd rigid body refinement: Similar geometries for A+D, B+ E and C+F

Table S7 Unit cell parameters for L-Nva at 320 K and L-Met at 430 K

Compound	L-Nva	L-Met
Temperature/K	320	430
Space group	*C*2	$P2_1$
a/Å	9.62(5)	9.57(2)
b/Å	5.24(3)	5.186 (11)
c/Å	15.37(8)	15.61(4)
β/°	112.2(2)	94.13(10)
V/Å^3	717(9)	773(5)
Z, *Z*’	4, 1	4, 2
N_{refl}	142	177

Data transformation for L-Nle at 100 K

Cell parameters:

a (Å)	9.5245(10)
b (Å)	5.2348(6)
c (Å)	14.6489(16)
β (deg)	98.741(9)
V (Å^3)	721.89(14)

Modulation vector: 2/3$\mathbf{a}^*$ – 1/6$\mathbf{c}^*$

Transformation to real indices expressed with respect to the superspace cell:

$$(hklm) \rightarrow (h + 2/3m, k, l - 1/6m)$$

Transformation to the supercell:

$$(h + 2/3m, k, l - 1/6m)\begin{bmatrix} 3 & 0 & -1 \\ 0 & 1 & 0 \\ 0 & 0 & 2 \end{bmatrix} = (HKL)$$

On hydrogen bonding in the crystal structures of hydrophobic amino acids in their enantiomeric form

The general construction principle for “hydrophobic” amino acid crystal structures, i.e. when there are no strong hydrogen-bond donors or acceptors in the side chains, is illustrated in Figure 10S (Görbitz *et al.*, 2009). A few exceptions are discussed in a recent review (Görbitz, 2015).

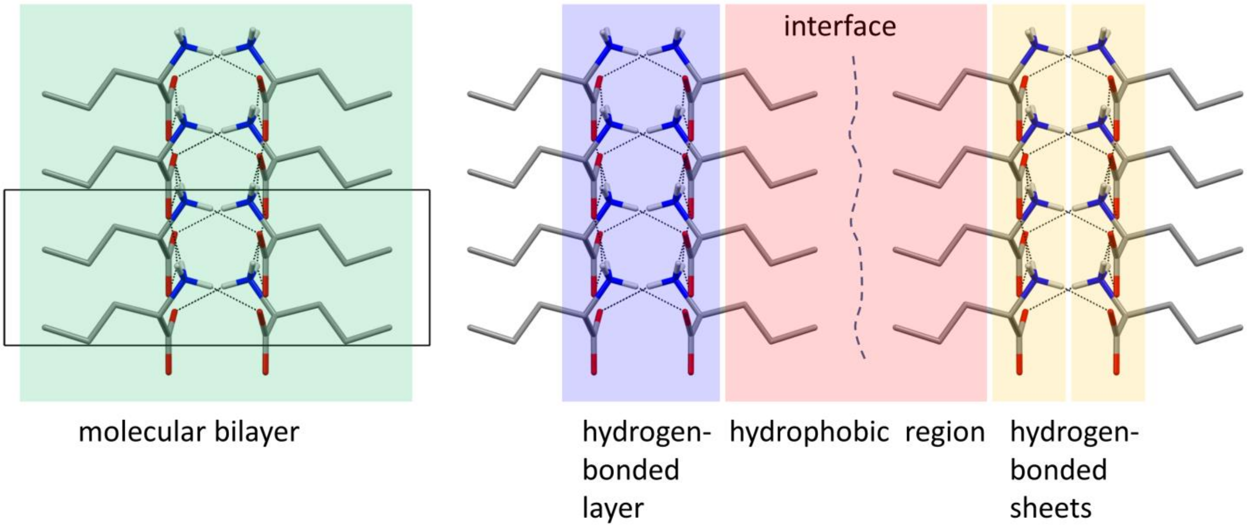

Figure S12 Typical features of the crystal structure of a hydrophobic amino acid seen in for L-Nva at 293 K, see text for details.

The crystal packing can be regarded as a systematic stacking of molecular bilayers (green shade). The side chains constitute the two surfaces of each bilayer and form hydrophobic regions (red shade) with a central interface between the bilayers. At the core of each bilayer is a hydrogen-bonded layer (blue shade) constructued from two sheets (orange shades). Two of the three amino hydrogen atoms form hydrogen bonds within a sheet while the third connects neigboring sheets.

Four types of sheets called L1, L2, L3 and Lx have been discerned in L-amino acids (Görbitz *et al.*, 2009), Figure 11S. The corresponding D-amino acids would form the mirror image D1, D2, D3 and Dx sheets.

Racemates and quasiracemates use only the L1 sheet, which is then paired with a D1 sheet to form a L1–D1 layer, and also form a fifth type of sheet called LD that contains amino acids of both hands (Görbitz *et al.*, 2009).

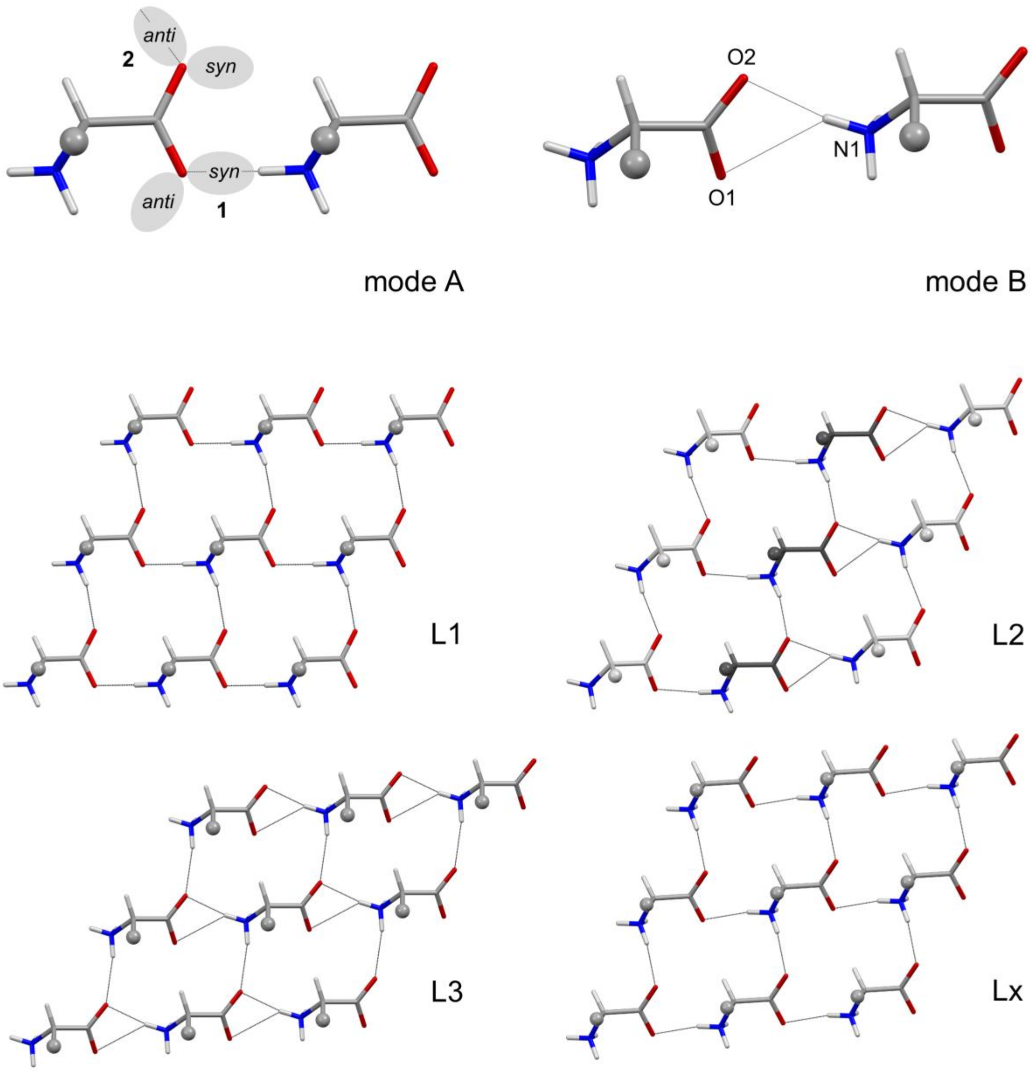

Figure S13 Hydrogen-bonded sheets in enatiomeric amino-acid structures. Side chains are shown as small spheres, the two independent molecules in the L2 sheets have carbon atoms of different colors.

One of the two hydrogen bonds within a sheet is accepted by a carboxylate *syn* lone pair (interaction 1), the second by an *anti* lone pair (interaction 2). The former comes in different versions: in mode A the hydrogen bond is two-centered with O1 as the acceptor,* while in mode B it is three-centered with H···O2 shorter than H···O1. The four sheets differ in the following way:

- L1 has only mode A, the number of molecules in the asymmetric unit (*Z*’) can be as low as 1.
- L2 has both modes, *Z*’ is 2.
- L3 has only mode B, *Z*’ is 1.
- Lx represents a hybrid between the A and B modes, *Z*’ can be as low as 1.

The *syn* hydrogen bond in Lx sheets can usually be considered as three-centered with H···O1 (much) shorter than H···O2. The L3 sheet has been found only for 5-methyl L-glutamate with CSD refcode GAVRAX (Wu *et al.*, 2005). Notably, L1 and Lx sheets may occur in structures with *Z*’ > 1, in which case all molecules in the asymmetric unit participate in very similar hydrogen bonds, but

* O1 is taken to be the carboxylate oxygen atom that gives O1–C1–C2–N1 torsion angle close to 0°.

differ with respect to side-chain conformations. L-Phe form I (Ihlefeldt *et al.*, 2014) and L-Abu (Görbitz, 2010) are two examples.

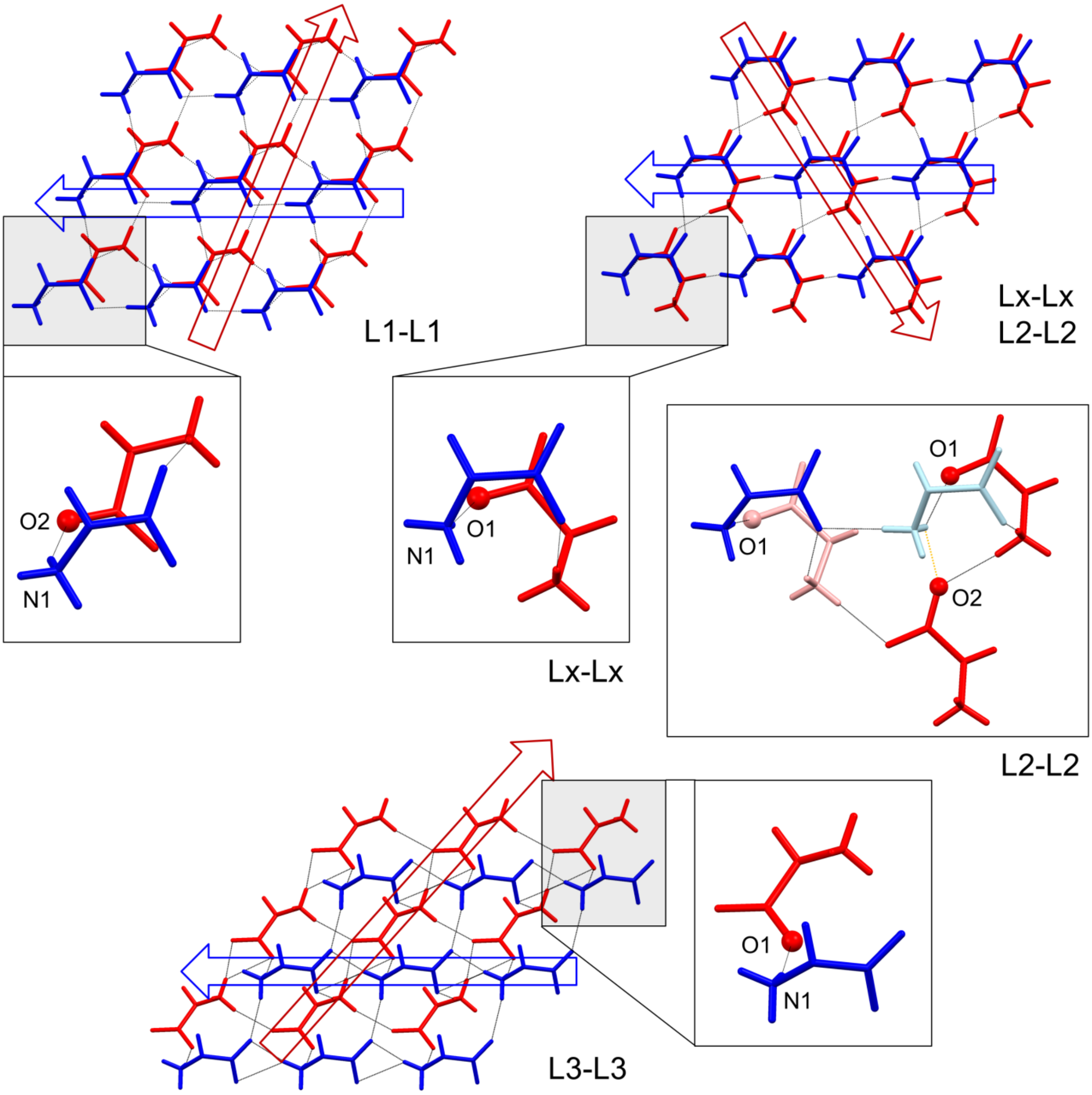

Figure S14 Hydrogen-bonded layers in enatiomeric amino acids. Side chains have been omitted, molecules in the top sheet are coloured in blue, molecules in the bottom sheet in red, see text for details.

Figure 12S compares the ways two individual sheets come together and form a hydrogen-bonded layer. The large open arrows give the directions of the N–H bond vectors in hydrogen bonds to carboxylate *syn* lone pairs for the upper (blue) and the lower (red) sheet. Direction vectors for the L2–L2 layer and the Lx–Lx layer (top right) are the same. It is important to note that although the difference between a L1 sheet and a Lx sheet in Figure 11S may seem insignificant, Figure 12S shows a 125° clockwise rotation of the red arrow from the L1–L1 pattern (top left) to the Lx–Lx pattern. Consequently, there are no borderline cases between these two.

The detailed views in Figure 12S (in boxes, rotated slightly to reduce overlap) focus on the hydrogen bonds connecting the sheets. In an L1–L1 layer O2 is the acceptor for this interaction, while it is O1 in an Lx–Lx layer. In a L2–L2 layer both molecule 1 (dark blue, dark red) and molecule

2 (light blue, light red) use O1 as the acceptor, but for molecule 2 the interaction is three-centered with the minor component (colored in orange) to O2. Like Lx–Lx, the L3–L3 layer is distinct from the L1–L1 in that O1, not O2, is the acceptor. Furthermore, while L1–L1, L2–L2 and Lx–Lx layers incorporate amino acid dimers (1–2 heterodimers for L2–L2), these are absent in the L3–L3 layer.

L2–L2 hydrogen-bonded layers occur for the common hydrophobic amino acids L-Val, L-Leu and L-Ile, and it has been shown by *ab initio* calculations (Görbitz *et al.*, 2009) that this pattern is inherently better (i.e. has lower energy) than the three alternatives with *Z*′ = 1. Amino acids with unbranched side chains, including L-Met (Dalhus & Görbitz, 1996a) and the monoclinic polymorph of L-Cys (Görbitz & Dalhus, 1996) account for four out of twelve known structures with L2–L2 layers (Görbitz, 2015), but they may also form Lx–Lx layers, as seen for e.g. L-Abu (Görbitz, 2010) and L-Nle (Torii & Iitaka, 1973). This suggests a fine energy balance between packing arrangements with Lx–Lx and L2–L2 hydrogen bonding patterns, where the less favorable hydrogen bonding interactions of the former may be counteracted by better hydrophobic stacking interactions (Görbitz *et al.*, 2009)
